# Oxidation of Styrene to Benzaldehyde Catalyzed by Schiff Base Functionalized Triazolylidene Ni(II) Complexes

**DOI:** 10.3390/molecules27154941

**Published:** 2022-08-03

**Authors:** Nasir S. Lawal, Halliru Ibrahim, Muhammad D. Bala

**Affiliations:** School of Chemistry & Physics, University of KwaZulu-Natal, Westville Campus, Private Bag X54001, Durban 4000, South Africa; lawalnasir70@yahoo.com (N.S.L.); ibrahimh@ukzn.ac.za (H.I.)

**Keywords:** styrene, Ni(II) triazolylidene complex, Schiff base, catalysis, oxidation

## Abstract

Four new Schiff base functionalized 1,2,3-triazolylidene nickel complexes, [Ni-(L_1_NHC)_2_](PF_6_)_2_; **3**, [Ni-(L_2_NHC)_2_](PF_6_)_2_; **4**, [Ni-(L_3_NHC)](PF_6_)_2_; **7** and [Ni-(L_4_NHC)](PF_6_)_2_; **8**, (where L_1_NHC = *(E)-3-methyl-1-propyl-4-(2-(((2-(pyridin-2-yl)ethyl)imino)methyl)phenyl)-1H-1*,*2*,*3-triazol-3-ium hexafluorophosphate(V)*, **1**, L_2_NHC = *(E)-3-methyl-4-(2-((phenethylimino)methyl)phenyl)-1-propyl-1H-1*,*2*,*3-triazol-3-ium hexafluorophosphate(V)*, **2**, L_3_NHC = *4*,*4′-(((1E)-(ethane-1*,*2-diylbis(azanylylidene))bis(methanylylidene))bis(2*,*1-phenylene))bis(3-methyl-1-propyl-1H-1*,*2*,*3-triazol-3-ium) hexafluorophosphate(V)*, **5**, and L_4_NHC = *4*,*4′-(((1E)-(butane-1*,*4-diylbis(azanylylidene))bis(methanylylidene))bis(2*,*1-phenylene))bis(3-methyl-1-propyl-1H-1*,*2*,*3-triazol-3-ium) hexafluorophosphate(V)*, **6**), were synthesised and characterised by a variety of spectroscopic methods. Square planar geometry was proposed for all the nickel complexes. The catalytic potential of the complexes was explored in the oxidation of styrene to benzaldehyde, using hydrogen peroxide as a green oxidant in the presence of acetonitrile at 80 °C. All complexes showed good catalytic activity with high selectivity to benzaldehyde. Complex **3** gave a conversion of 88% and a selectivity of 70% to benzaldehyde in 6 h. However, complexes **4** and **7**–**8** gave lower conversions of 48–74% but with higher (up to 90%) selectivity to benzaldehyde. Results from kinetics studies determined the activation energy for the catalytic oxidation reaction as 65 ± 3 kJ/mol, first order in catalyst and fractional order in the oxidant. Results from UV-visible and CV studies of the catalytic activity of the Ni-triazolylidene complexes on styrene oxidation did not indicate any clear possibility of generation of a Ni(II) to Ni(III) catalytic cycle.

## 1. Introduction

The selective oxidation of styrene to benzaldehyde is an interesting research area for academia and industry owing to the versatile role of the carbonylic group as a building block leading to a variety of products for perfumery and food processing industries. Benzaldehyde, one of the oxidation products of styrene, is the second most important aromatic molecule (after vanillin) in the production of perfumes, pharmaceuticals, dyestuffs and agrochemicals. It is commercially obtained as a by-product of the catalytic oxidation of toluene to benzoic acid or by the hydrolysis of benzyl chloride [1]. However, the former process is prone to selectivity issues while the latter suffers from traces of chlorine waste which is not acceptable in perfumes and pharmaceutical chlorine-free products [2]. Thus, an alternate method that is selective and free of chlorine contamination is desirable.

The end of the 20th century brought new ways of doing chemistry, simply termed ‘green chemistry’. Keeping to the principles of this concept has motivated chemists to design new methods of running reactions that are often cheaper, safer and environmentally benign [3]. Oxidation of styrene to benzaldehyde is one in which green oxidants such as hydrogen peroxide, molecular oxygen, and air are preferred over harsh oxidizing agents such as KMnO_4_ and K_2_Cr_2_O_7._ The key advantage of these oxidants is that they are cheap, and the only by-product of the reaction is H_2_O. Moreover, the reactions are often very selective in the presence of catalysts, thereby eliminating unwanted side products at improved process efficiencies [4].

Despite the abundance of well-established catalyst systems for producing industrially viable products from cheap hydrocarbon sources [5], teething problems associated with their widespread adaptation have led to setbacks in their application and have remained research challenges for academia and industry. Adapting the 12 principles of green chemistry in synthesis and catalyst design has led to improved catalyst systems. These systems function without the need for aggressive chlorinated oxidants or the use of stoichiometric amounts of oxidants. Additionally, the selectivity to products is improved with a high atom economy. Furthermore, research in the transition-metal-catalyzed oxidation of hydrocarbons has shown that the choice of solvent, ligand and oxidant influence the catalysis and type of products obtainable [6,7]. Hence, the use of multidentate and chelating ligands [8], protic solvents (isopropanol, ethanol, methanol) [9], and green oxidants (O_2_, H_2_O_2_, or tert-butyl hydroperoxide (TBHP) [3], have all been explored due to their attributes of low cost, environmental friendliness and the potential for driving reactions at high catalytic efficiencies.

Transition metal complexes of Schiff base ligands have been extensively studied because of their ease of synthesis, remarkable electronic tunability, and their potency when applied as catalysts in oxidation reactions. In this context, Schiff base metal complexes of manganese(II) [10,11,12], vanadium(IV) [13,14], cobalt(II) [15,16], nickel(II) [14,16,17,18], iron(II) [15,19] and copper(II) [14,20,21] have previously been tested for their catalytic activity in styrene oxidation reactions. Each catalyst system recorded a preference for either of the two oxidation products (i.e., styrene oxide or benzaldehyde), and moderate to good yields were generally reported. Other non-Schiff base complexes of Ni have also shown interesting selectivity in catalyzing the oxidation of styrene to styrene oxide using oxygen as an oxidant [22]. Despite the reported efficiencies of NHC-based metal complexes as catalysts for various organic transformation reactions, reported works on their use as catalysts for the oxidation of styrene are few [23], and those based on imino-functionalized nickel complexes are novel.

Most of the proposed styrene oxidation reaction mechanisms involve high oxidation states of the metal ions during the catalytic cycle [23,24,25]. Hence, there is still a strong need for ligands capable of stabilizing these electronically demanding metal ions. It is well-documented that in comparison to imidazolium-based NHCs and other two-electron donor atoms (N, P, O), the triazolylidenes are stronger donor ligands [26]. They are also more capable of stabilizing high oxidation state metal ions in solution and could provide the needed stability to the metal centre during the oxidation cycle. Based on this hypothesis, we herein present for the first time the application of new nickel Schiff base functionalized triazolylidene (NHC) complexes for the selective catalytic oxidation of styrene to benzaldehyde under mild and environmentally compatible reaction conditions.

## 2. Materials and Methods

All reagents and substrates were purchased from Sigma Aldrich, Merck (Pty) Ltd. (Lethabong, South Africa) and used as received. Solvents (acetonitrile, dichloromethane, methanol and diethyl ether) were purchased from Merck and purified using a commercially available MBraun MB-SP Series solvent purification system equipped with activated alumina columns. Unless otherwise stated, all syntheses were performed under a nitrogen atmosphere using standard Schlenk techniques. NMR spectra were recorded on a Bruker Avance 400 MHz spectrometer operated at ambient temperature with δ values reported in ppm referenced to Me_4_Si as the internal standard for both ^1^H and ^13^C NMR data. Infrared spectra were recorded on a Perkin Elmer universal ATR Spectrum 100 FT-IR spectrometer. Mass spectrometry and elemental analysis (where applicable) were recorded on Waters Micromass LCT Premier TOF MS-ES^+^ and ThermoScientific Flash 2000 Elemental Analyzer, respectively. Melting points were recorded using an Electrothermal 9100 melting point apparatus. GC analysis was conducted on an Agilent Technology 6820 GC System equipped with a flame ionization detector (FID) and an Agilent ZB-Wax column with a length of 30 m, an inner diameter of 0.25 mm and a thickness of 0.25 mm. All ligand precursors, **1**–**2** and **5**–**6** (L_1_NHC–L_4_NHC) used to synthesize complexes **3**–**4** and **7**–**8** were reported elsewhere [27,28,29].

### 2.1. General Synthesis of Nickel Triazolylidene Complexes

All the complexes, **3**–**4** and **7**–**8**, were synthesized via the silver oxide transmetalation route [30]. A general description is as follows: In a 15 mL Schlenk tube, a weighed amount of the respective Schiff base functionalized 1,2,3-triazolylidene ligand precursor and 2 mole equivalent of silver(I) oxide in dry dichloromethane was stirred at room temperature for 4 h under nitrogen. The mixture was filtered through a short bed of Celite, and the yellow filtrate was added to a methanolic solution of ½ mole equivalent (for complexes **3**–**4**) or 1 mole equivalent (for complexes **7**–**8**) of Ni(diglyme)Cl_2_. The resulting mixture was then continuously stirred at room temperature for 12 h under a dinitrogen atmosphere. Subsequent filtration and removal of all volatiles in vacuo gave respective solids that were washed with ether (10 mL × 3) to afford the Ni(II)NHC complexes **3**–**4** and **7**–**8** in good to excellent yields.

*[Ni-(L*_1_*NHC)*_2_*](PF*_6_*)*_2_, **3**

The complex was synthesized using the following quantities of starting materials; Ligand precursor, **1**, (0.479 g, 1 mmol), Ag_2_O (0.484 g, 2 mmol) and Ni(diglyme)Cl_2_ (0.109 g, 0.5 mmol). Light green solid. Yield 0.452 g, 89%. Mp = 92–94 °C. ^1^H-NMR (DMSO, 400 MHz): δ 0.88 (3H, t, *J* = 7.2 Hz, CH_3 Pr_), 1.02 (3H, t, *J* = 6.6 Hz, CH_3 Pr_), 1.70 (2 × 2H, s, C_2_H_4_-), 1.94 (2 × 2H, m, C_2_H_2 Pr_), 3.52 (2 × 2H, s, C_2_H_2_-N=), 3.95 (3H, s, CH_3_), 4.08 (3H, s, ‘CH_3_), 4.62 (2 × 2H, t, *J* = 7.8 Hz, C_2_H_2_-N= _Pr_), 7.30 (4 × 1H, d, *J* = 22.7 Hz, Ar), 7.66 (4 × 1H, t, *J* = 14.1 Hz, Ar′), 7.85 (2 × 2H, t, *J* = 15.7 Hz, Py, Py′), 8.04 (2 × 1H, d, *J* = 12.7 Hz, Py), 8.44 (2H, d, *J* =59.7 Hz, Py′), 9.10 (1H, s, CH=N _imine_), 9.90 (1H, s, ‘CH=N _imine_). ^13^C-NMR (DMSO, 100.6 MHz): δ 10.31, 22.20, 38.03, 54.75, 130.13, 131.79, 132.35, 132.45, 132.75, 134.34, 192.32. IR (ATR, cm^−1^): 3273 (O-H), 2173 (C-H, sp^2^), 1656 (C=N), 1607 (C=C), 1161 (N=N), 836 (${\mathrm{PF}}_{6}^{-}$), 767 (Ar C-H), 556 (M-N). MS-ES^+^: *m/z* (%) 393 (1.8) [(Ni+L_1_NHC+H)]^+^, 334 (100) [(L_1_NHC+H)]^+^.

*[Ni-(L*_2_*NHC)*_2_*](PF*_6_*)*_2_, **4**

The complex was synthesized using the following quantities of starting materials; Ligand precursor, **2**, (0.478 g, 1 mmol), Ag_2_O (0.484 g, 2 mmol) and Ni(diglyme)Cl_2_ (0.109 g, 0.5 mmol). Light green solid. Yield 0.425 g, 84%. Mp = 122–124 °C. ^1^H-NMR (CH_3_CN, 400 MHz): δ 0.91 (3H, s, CH_3 Pr_), 1.14 (3H, s, ‘CH_3 Pr_), 1.95 (2 × 2H, s, -CH_2_- _Pr_), 2.82 (2H, s, CH_2_CH_2−_), 3.33 (2H, s, ‘CH_2_CH_2__−_), 3.70 (2 × 3H, s, CH_3_) 3.95 (2H, s, C_2_H_2_-N=), 4.12 (2H, s, ‘C_2_H_2_-N=), 4.60 (2 × 2H, s, C_2_H_2_-N= _Pr_), 7.41 (2 × 4H, m, Ph), 7.78 (2 × 1H, s, Ph), (2 × 4H, m, Ar), 8.50 (1H, s, CH=N _imine_), 10.02 (1H, s, ‘CH=N _imine_). ^13^C-NMR (CH_3_CN, 100.6 MHz): δ 10.71, 23.55, 38.90, 56.44, 121.64, 129.16, 129.24, 129.37, 129.50, 129.58 133.50, 133.66, 134.93, 135.32, 193.06. IR (ATR, cm^−1^): 3204 (O-H), 2165 (C-H, sp^2^), 1602 (C=N), 1578 (C=C), 1200 (N=N), 842 (${\mathrm{PF}}_{6}^{-}$), 778 (Ar C-H), 557 (M-N). MS-ES^+^: m/z (%) 757 (5.0) [(M+CH_3_OH+2H)]^+^.

*[Ni-(L*_3_*NHC)](PF*_6_*)*_2_, **7**

The complex was synthesized using the following quantities of starting materials; Ligand precursor, **5**, (0.387 g, 0.5 mmol), Ag_2_O (0.242 g, 1 mmol) and Ni(diglyme)Cl_2_ (0.109 g, 0.5 mmol). Light brown solid. Yield 0.378 g, 91%. Mp = 81–83 °C. ^1^H-NMR (DMSO, 400 MHz): δ 1.07 (3H, t, *J* = 6.9 Hz, CH_3 Pr_), 1.12 (3H, t, *J* = 6.3 Hz, ‘CH_3 Pr_), 2.03 (2 × 2H, m, -CH_2_- _Pr_), 3.69 (2H, t, *J* = 6.9 Hz, N-CH_2_CH_2_-N), 3.94 (2H, t, *J* = 8.3 Hz, N-CH_2_CH_2_-N), 4.13 (2 × 3H, s, CH_3_), 4.69 (2 × 2H, t, *J* = 6.9 Hz, C_2_H_2_-N= _Pr_), 7.97 (4 × 1H, m, Ar), 8.05 (2 × 1H, d, *J* = 7.2 Hz, Ar′), 8.11 (2 × 1H, d, *J* = 7.3 Hz, Ar′), 9.17 (1H, s, CH=N _imine_), 9.98 (1H, s, ‘CH=N _imine_). ^13^C-NMR (DMSO, 100.6 MHz): δ 10.67, 22.70, 24.68, 38.43, 49.10, 55.56, 65.72, 123.70, 131.22, 131.31, 131.41 132.83, 133.00, 133.22, 133.43, 134.87, 136.60, 140.43, 141.32, 142.25. IR (ATR, cm^−1^): 3272 (O-H), 2165 (C-H, sp^2^), 1622 (C=N), 1582 (C=C), 1201 (N=N), 852 (${\mathrm{PF}}_{6}^{-}$), 772 (Ar C-H), 557 (M-N). MS-ES^+^: m/z (%) 575 (22.1) [(M+CH_3_OH+2H)]^+^

*[Ni-(L*_4_*NHC)](PF*_6_*)*_2_, **8**

The complex was synthesized using the following quantities of starting materials; Ligand precursor, **6**, (0.401 g, 0.5 mmol), Ag_2_O (0.242 g, 1 mmol) and Ni(diglyme)Cl_2_ (0.109 g, 0.5 mmol). Light brown solid. Yield 0.408 g, 95%. Mp = 106–108 °C. ^1^H-NMR (DMSO, 400 MHz): δ 0.87 (3H, t, CH_3 Pr_), 1.02 (3H, t, ‘CH_3 Pr_), 1.30 (2 × 2H, s, NC-CH_2_CH_2_-CN), 1.92 (2 × 2H, s, -CH_2_- _Pr_), 3.93 (2 × 2H, d, *J* = 23.4 Hz, N-CH_2_-C-C-CH_2_-N), 4.11 (2 × 3H, s, CH_3_), 4.59 (2 × 2H, s, C_2_H_2_-N= _Pr_), 7.75 (4 × 1H, m, Ar), 8.24 (4 × 1H, m, Ar), 9.10 (1H, s, CH=N _imine_), 9.93 (1H, s, ‘CH=N _imine_). ^13^C-NMR (DMSO, 100.6 MHz): δ 10.77, 22.77, 28.41, 38.16, 49.15, 55.06, 60.62, 120.90, 129.78, 131.25, 132.57, 133.38, 135.62, 142.05, 159.99. IR (ATR, cm-1): 3350 (O-H), 2029 (C-H, sp^2^), 1686 (C=N), 1606 (C=C), 1168 (N=N), 840 (${\mathrm{PF}}_{6}^{-}$), 768 (Ar C-H), 556 (M-N). MS-ES^+^: *m/z* (%) 657 (3.3) [(M+CH_3_OH+Na)]^+^.

### 2.2. General Procedure for Styrene Oxidation

Catalytic oxidation of styrene with H_2_O_2_ (30% *v*/*v*) as oxidant was carried out in acetonitrile substrate solution (3 mL), initially measured into a 15 mL Schlenk tube connected to a condenser. Optimization of reaction conditions for the catalytic oxidation of styrene was studied by varying the following parameters:

(i) Styrene: hydrogen peroxide molar ratios from 1:3 to 1:10,
(ii) Reaction temperatures between 40 and 80 °C,
(iii) Catalyst concentration from 0.5 to 2 mol%.

Aliquots of 0.1 mL were taken with a hypodermic syringe from the reaction mixture at specific time intervals as the reaction progressed, filtered through a short plug of cotton wool and directly analyzed by gas chromatography (GC), equipped with a ZB-Wax capillary column (30 m × 0.25 mm internal diameter) and a flame ionization detector (FID). Product yield was quantified using the external standard method, and percentage conversion was recorded (average of two runs within ±5%).

### 2.3. UV–Vis Analysis

UV–Vis analysis was conducted on Shimadzu UV–Vis-NIR Spectrophotometer UV-3600. In a 3 mL cuvette, an acetonitrile solution of **3** (0.04 mM, 2 mL) was scanned from 200–700 nm wavelength. 0.2 mL of 30% H_2_O_2_ (0.4 mM) was added into the cuvette and scanned immediately; then scanned again at time intervals of 5 min and 1 h. Subsequently, 0.2 mL of styrene was added to the cuvette mixture and monitored for 6 h.

### 2.4. Cyclic Voltammetry (CV) Analysis

The CV analyses were performed in DMSO with 1M TBu_4_PF_6_ as a working electrolyte using Metrohm 797 potentiostat and a three-electrode system consisting of glassy carbon as a working electrode, a platinum wire as the reference electrode and an Ag/AgCl system as a counter electrode. All scans were performed after purging with a stream of nitrogen gas. Background checks were performed in the range of −1.2 V–1.2 V in DMSO and 1M TBu_4_PF_6_. Scan rates of 100, 250 and 500 mV/s were conducted in a potential window of −1.2 to 0.2 V.

## 3. Results and Discussion

### 3.1. Synthesis and Spectroscopic Characterization

**Scheme 1:**
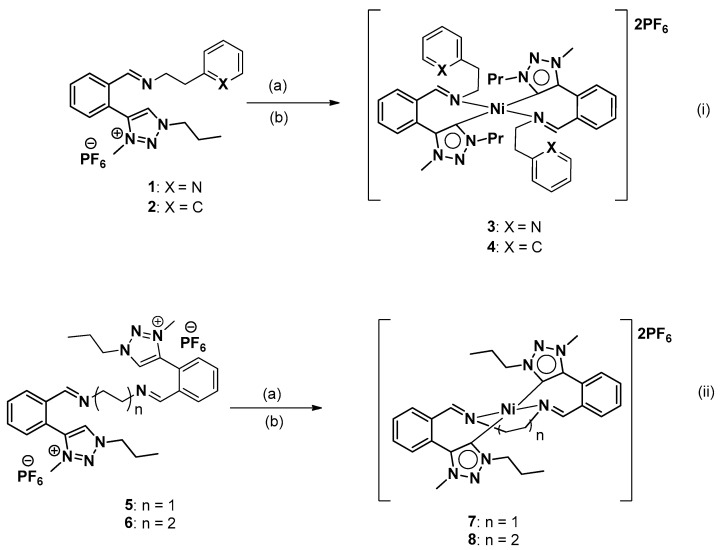
Synthesis of Schiff base functionalised triazolylidene Ni(II) complexes; (a) Ag_2_O, DCM, N_2_, stir, RT, 4 h. (b) Ni(diglyme)Cl_2_, MeOH, N_2_, stir, RT, 12 h; (**i**) functionalized mono carbene ligand frame, (**ii**) functionalized dicarbene ligand frame.

The synthesis and full characterization of the triazolylidene ligand precursors **1**–**2** and **5**–**6** (Scheme 1) were reported elsewhere [27,28,29]. All nickel complexes were synthesized via transmetalation from corresponding in situ generated Ag-NHC complexes, using Ni(diglyme)Cl_2_ as the metal source (Scheme 1i,ii). The afforded complexes **3**–**4** and **7**–**8** were obtained in good to excellent yield as light green or brown solids and were characterized by IR, MS and NMR spectroscopy (see Supplementary Materials).

**Figure 1:**
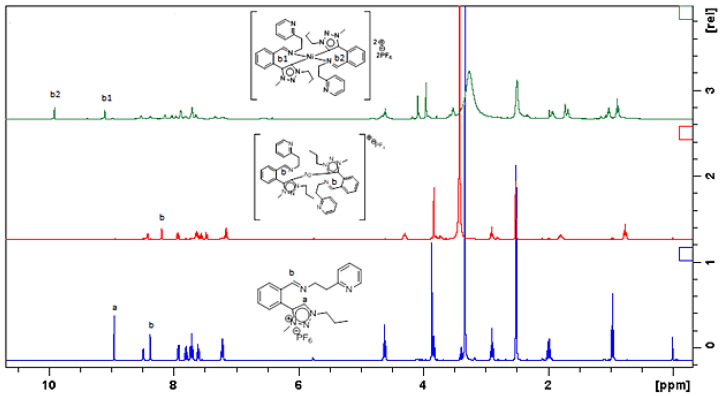
Stacked ^1^H NMR spectra showing the disappearance of the carbene (C_5_-H) proton from the ligand precursor (blue line, position a at 8.9 ppm) to silver complex (red line) and on the formation of the nickel complex, **1** (green line).

Preliminarily, the ^1^H NMR spectra of the in situ generated Ag-NHC pre-complex from each ligand precursor indicated the complete disappearance of the triazolium proton and subsequent formation of Ag-carbene bonds (ESI and Figure 1). This, coupled with observed shifts and broadening in the splitting patterns of other proton signals compared to the positions in the spectra of the triazolylidene precursors, confirmed the formation of carbene–metal bonds in the Ag-NHC complexes. Other changes were observed during transmetalation to Ni(II), which include distinct colour changes, precipitation and solubility differences. These physical observations gave a preliminary indication of the successful transmetalation of the NHC ligands from Ag(I) to Ni(II). The IR spectra of complexes **3**–**4** and **7**–**8** showed very strong and sharp absorptions at 1656–1578 cm^−1^, characteristic of Ni-NHC complexes [31] assigned to the C=N stretching vibration of the metal centre bound imine donor.

As shown in Figure 1 and the supporting information, all the expected proton peaks are accounted for in the ^1^H NMR spectra of the Ni-triazolylidene complexes **3**–**4** and **7**–**8**. The broadness of the proton resonance signals in the respective ^1^H NMR spectra of the complexes is similar to that reported for analogues complexes and showed clear evidence of ligand coordination to the metal via the imine and carbenoid donors [32,33]. Interestingly, the ^1^H NMR spectra of both **3** and **4** gave a very similar distribution of protons, indicating a similar mode of coordination of the donor groups to the metal. These include the disappearance of the triazolium C5 proton (*a*, in Figure 1, ESI) and a significant downfield shift for the imine (HC=N) protons. Hence, coordination of each ligand to the metal centre in **3** and **4** is proposed via the carbene C-atom and imine N-atom. It is worthy to note that complex **3** bears a pyridyl side group that has the potential to bind to a metal centre. However, the available characterization data indicate that the imine N atom is preferentially bound to the Ni(II) centre. This is justifiable based on Pearson’s hard and soft acids and bases (HSAB) principle. A hard imine donor will preferentially bind to a hard Ni(II) centre with a lesser chance of binding for the softer heterocyclic pyridyl-N donor as its electron density is delocalized in the aromatic sextet [34]. This supports the proposal that the ligand coordination to the Ni(II) is via the carbene C and the imine N atoms [35,36,37,38]. Despite the structural similarity between **3** and **4**, there is still unique potential hemilability in the structural motif of **3**, and it is most likely the reason for its good catalytic activity (discussed later). On the other hand, both **7** and **8** displayed similar ^1^H NMR spectral splitting patterns to compounds **3** and **4**. The main difference was observed in the alkyl region, which is accounted for in **7** (C_2_H_4_) and **8** (C_4_H_8_) as two separate singlets. The symmetric nature of the ligands in complexes **7** and **8** is responsible for the proposed square planar geometry around each Ni(II) centre with the ligands bound in a tetradentate CNNC coordination mode.

However, the coordination of the two ligands in complexes **3**–**4** and **7**–**8** is best described as distorted square planar. This is because of the observance of two distinct imines (HC=N) signals (b1 and b2 in Figure 1, see ES) in the proton NMR spectra of each nickel complex that was absent in the corresponding spectra of the Ag complex or the ligand precursor (b in Figure 1). The disappearance of only the carbene proton in the Ag(I) complex can be explained based on the HSAB principle, such that it is conceivable that the hard imine (HC=N) will not bind to the soft Ag(I) metal centre. Hence, the Ag(I) metal binds only to the ligand precursors’ carbene (C5 atom) in a linear coordination mode typical of *d*^10^ Ag^+^ complexes.

### 3.2. Peroxidative Conversion of Styrene Catalyzed by Complexes ***3***–***4*** and ***7***–***8***

The synthesized complexes **3**–**4** and **7**–**8** were utilized for the catalytic oxidation of styrene **9** (Table 1). The reaction conditions reported by Liu et al. [18] were adopted for the optimization of catalytic conditions. Hence, as presented in Table 1, blank reactions with H_2_O_2_ only (i.e., no catalyst, entry 1) and with complex **3** as representative catalyst (i.e., no oxidant, entry 2) were conducted at the beginning of the study to respectively establish independent influences of the oxidant and complex on the catalyzed reaction. The reaction in the absence of a catalyst yielded 22% conversion of the substrate with 97% selectivity to benzaldehyde, **10** (Table 1; entry 1), while no conversion was recorded in the absence of the oxidant even though a catalyst was added (Table 1; entry 2). This is not surprising as many reports on oxidation reactions have established the absolute need for the oxidant [14,17,18,39]. A combination of both oxidant and catalyst gave 77% conversion in 3 h (Table 1; entry 3). However, an increase in reaction time does not result in a significant increase in percentage conversion, with only a 10% increase recorded when the reaction time was extended to 6 h (Table 1; entry 4). These initial observations indicated the need for a catalyst/oxidant combination based on the Ni-NHC complexes as pre-catalysts for the oxidation of the styrene.

The metal precursor, Ni(diglyme)Cl_2,_ was also tested as a baseline for the Ni-triazolylidene complex and gave only 35% conversion after 6 h (Table 1; entry 5). This confirmed that the catalyst’s efficiency is enhanced by the stabilizing effect of the ligand bound to the metal centre, which invariably controls the oxidation state of the metal during the catalytic reaction [40,41,42]. However, 26% conversion to styrene oxygenates was detected when only the ligand precursor, **1**, was used as a metal-free catalyst (Table 1; entry 6). This emphasizes the importance of the metal centre in the catalyst system and, invariably, the required oxidation state of the metal in catalyzing the oxidation reaction.

Based on these initial catalytic results, the remaining Ni-triazolylidene complexes (**4**, **7** and **8**) were screened to determine the most active pre-catalyst for the styrene oxidation (Table 1; entries 7–12). The results showed a trend of reactivity in the following order: **3** > **4** > **8** > **7**. Although complex **3** gave the best conversion of 88% after 6 h of reaction compared to the other complexes, complexes **4**, **7** and **8** had better selectivities to benzaldehyde, **10** (> 90%), with only traces of acetophenone, **11** and styrene oxide, **12**. Relating the trend in catalytic activity to the structures of the complexes; the major structural difference between the most active catalyst **3**, and the other catalysts (**4**, **7** and **8**) is the pyridyl side group in its framework, which has the potential to enhance its stability as a secondary or hemilabile donor arm during catalysis hence leading to better substrate conversion. Similar trends have been noted for highly active catalysts bearing related structural motifs [43,44]. However, the catalytic efficiencies of complexes **7** and **8** in terms of selectivity to benzaldehyde are similar to the trends observed in Ni(II) complexes bearing sterically modified linear tetradentate N4 ligands reported by Sankaralingam et al. [45]. They opined that catalyst selectivity is largely dependent on the ligand donor groups. Similarly, the Ni(II) centres in **7** and **8** are meridionally coordinated by the two carbenoid and two imine donors of the ligands, thereby offering only one binding site in the axial position for the oxidant to attack the substrate leading to the moderate selectivities and TONs recorded.

**Figure 2:**
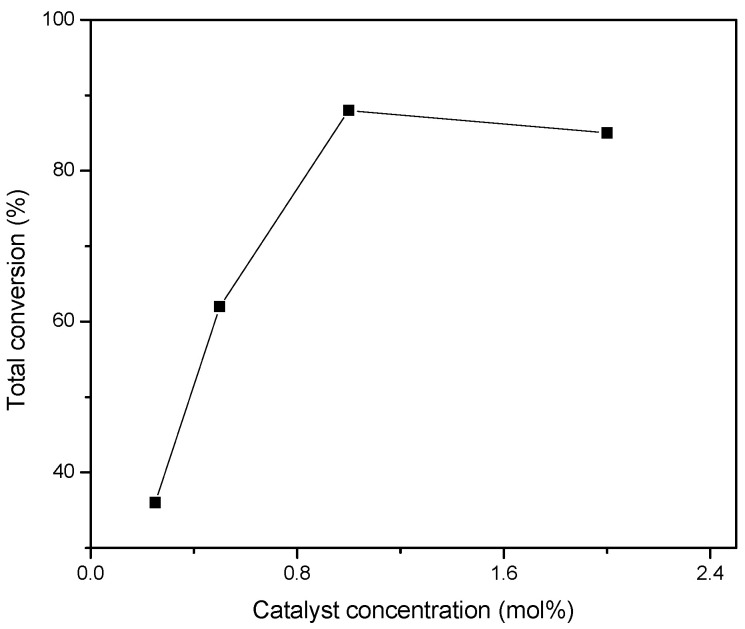
Total conversion of styrene to its oxygenates catalyzed by **3**, at varying catalyst concentrations.

Other reaction conditions such as catalyst concentration, amount of oxidant, temperature and reaction time were optimized using the most active complex, **3**, as the catalyst. Figure 2 presents the total conversion of styrene to benzaldehyde, and other oxygenates catalyzed by complex **3** at varying mole concentrations of the pre-catalyst. The total conversion to oxygenates is directly proportional to the catalyst concentration up to a maximum of 1.0 mol%, beyond which a plateau was observed at 2.0 mol%. At the most productive catalyst concentration based on complex **3**, 88% conversion of styrene was observed with 70% benzaldehyde selectivity. Thus, 1 mol% of the catalyst was used for the remainder of the study.

**Figure 3:**
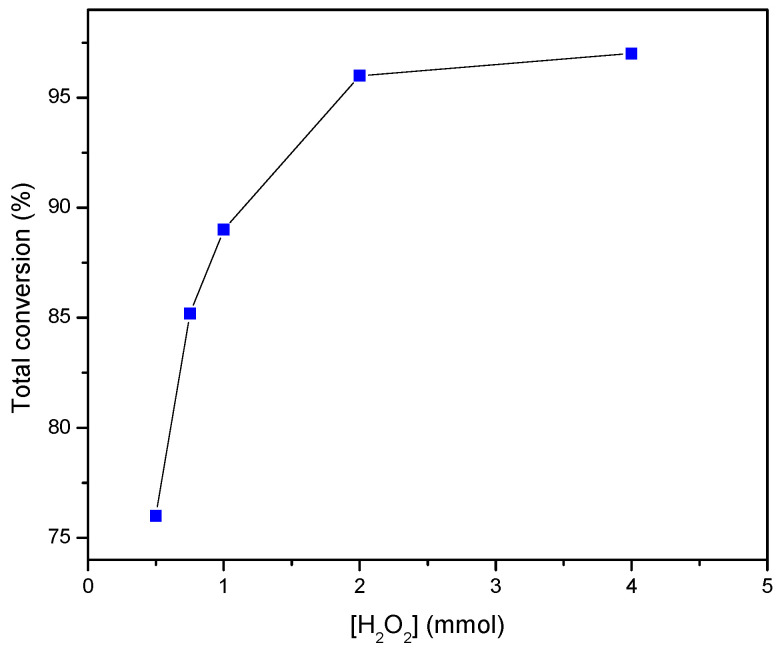
Total conversion of styrene to its oxygenates catalyzed by **3** at varying H_2_O_2_ concentrations.

Next, the effect of oxidant concentration on the catalytic system was determined, and the results are illustrated in Figure 3. The most productive conversion of styrene to its oxygenates was observed at 5–10 molar equivalents concentration of the oxidant, which is similar to values reported for other Ni(II) catalyst systems applied for oxidation reactions [14,18].

**Figure 4:**
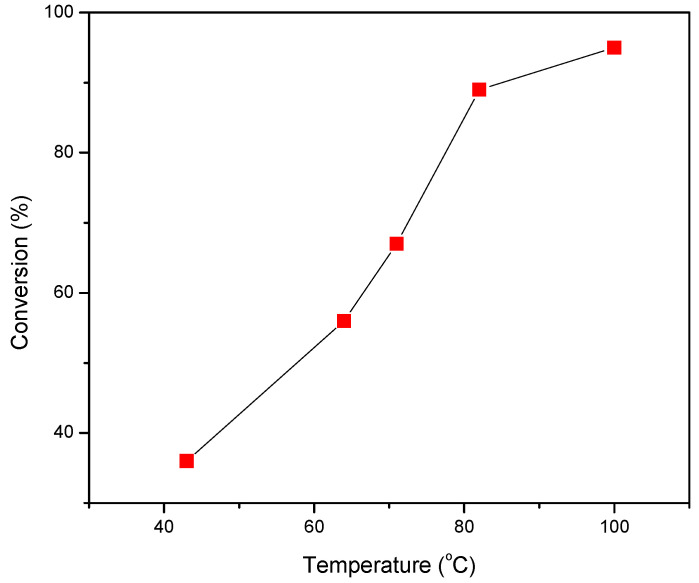
Temperature profile for the total conversion of styrene to its oxygenates catalyzed by complex **3**.

To determine the effect of thermal activation on the oxidation process, the temperature of the reaction was varied, and the results are presented in Figure 4, which shows a direct increase in styrene conversion to the oxygenates with an increase in temperature from 43–100 °C. This is in line with previous literature reports on styrene oxidation using acetonitrile as a solvent which reported 80 °C as the optimum temperature [14,17,18].

**Figure 5:**
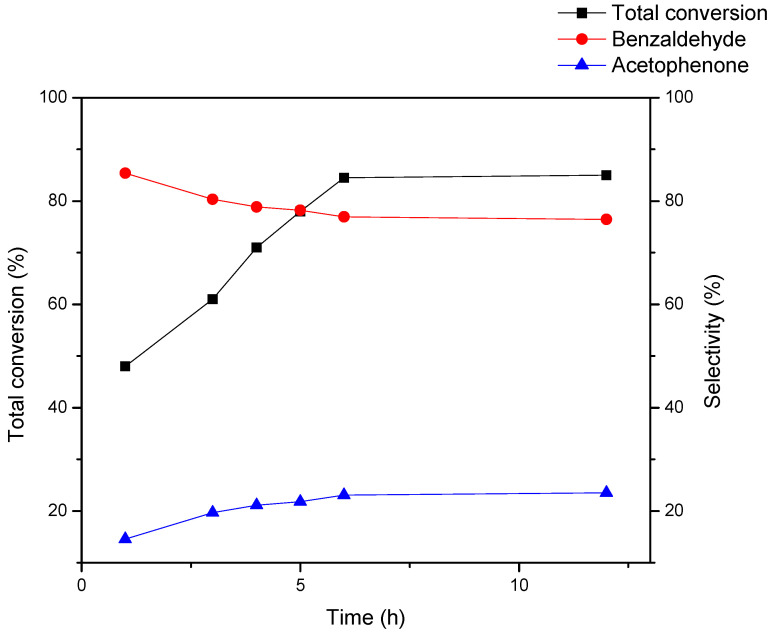
Time dependence profile and selectivity for the oxidation of styrene catalyzed by **3**.

Finally, to determine the optimum reaction time, the reaction was monitored over a 12 h period with sampling at regular intervals. The results presented in Figure 5 indicate that completion of the reaction occurs at 6 h, after which there is no significant increase in conversion. This is similar to observed trends for the oxidation of styrene in the literature, which were mostly conducted within 6 h [14,17]. Hence, with these results, the optimized reaction conditions for Ni(II) NHC complex catalyzed oxidation of styrene are summarised as: 0.2 mmol of styrene (substrate), 0.002 mmol of **1** (catalyst), 1.0 mmol of 30% H_2_O_2_, reflux at 80 °C for 6 h in 3 mL acetonitrile solvent.

Table 2 compares the results obtained for nickel complex **3** with other homogeneous catalytic nickel systems reported in the literature to contextualize the current studies. Analysis of the various data revealed that either H_2_O_2_ or TBHP as oxidants gave good conversions within 3–6 h. NiLa (Table 2; entry 3) gave the highest value in terms of benzaldehyde’s selectivity. All the reported Ni(II) complexes targeted for styrene oxidation (Table 2; entries 3–5) are characterized by a square planar geometry. Specifically, entries 3 and 4 represent tetradentate salen-type Schiff base complexes with ONNO donor atoms, while entry 5 represents a dihydroindolone ligand coordinated to the metal via OOOO donor atoms. When these are compared to results obtained in the current work (entries 1–2), the strong carbene–metal bond coupled with the imine N-donor bound in a chelating mode gave desired stability to the metal centres. Hence, much better substrate conversions were recorded compared to the mentioned traditional Schiff base containing complexes.

### 3.3. Determination of the Kinetics of Styrene Oxidation Catalyzed by Complex ***3***

**Figure 6:**
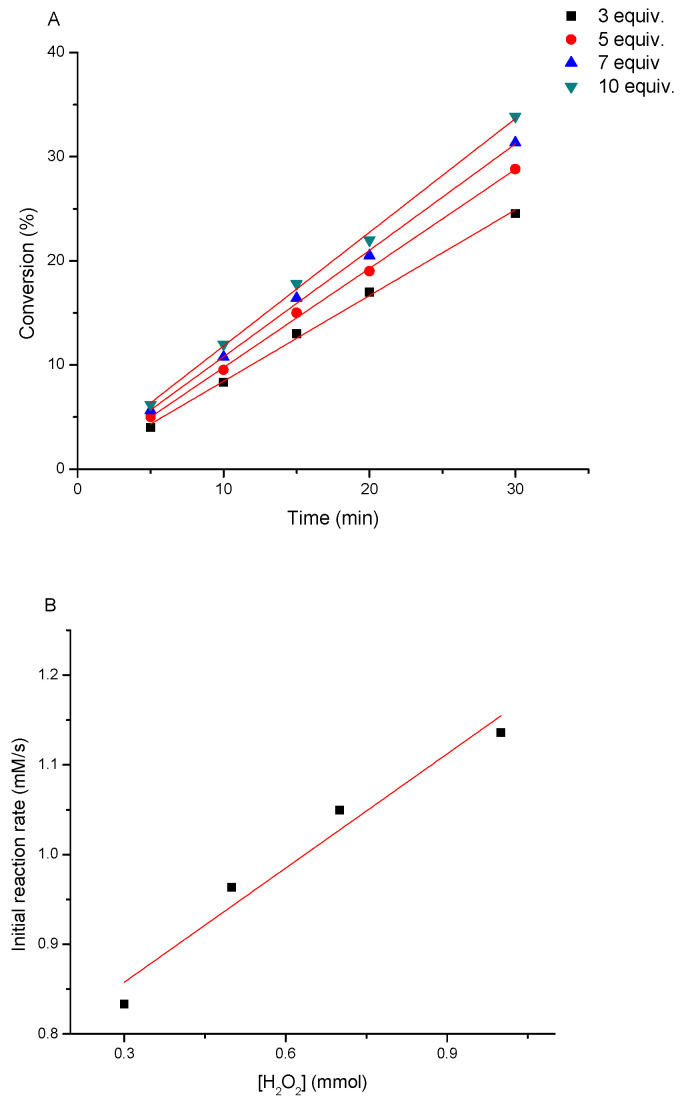
Influence of H_2_O_2_ concentration on (**A**) styrene conversion and (**B**) initial reaction rate of styrene oxidation. Reaction conditions: T = 80 °C; [styrene] = 0.2 mmol; [catalyst] = 0.002 mmol; acetonitrile = 3 mL.

In an attempt to shed some light on the kinetics of the oxidation process, the initial rate method was applied. In all the experiments, rates of styrene oxidation were evaluated graphically based on the amount of styrene converted as a function of time. The slope of the graph was used to determine the initial rates [46,47]. The initial concentrations of catalyst, styrene, acetonitrile and the reaction temperature were all kept constant, whilst the initial concentration of hydrogen peroxide was varied. The results presented in Figure 6 show that the initial reaction rate increased almost linearly with hydrogen peroxide concentration resulting in a first-order dependence which in context agrees with the reports of Saux and coworkers [47].

**Figure 7:**
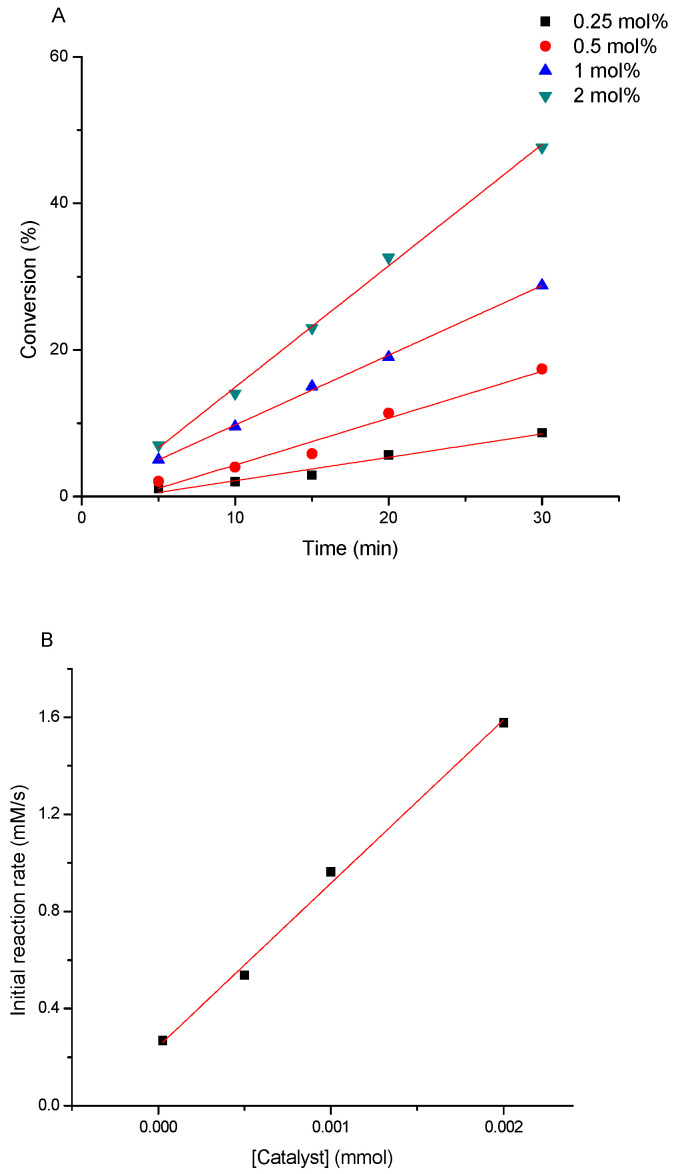
Influence of catalyst concentration on (**A**) styrene conversion and (**B**) initial reaction rate of styrene oxidation. Reaction conditions: T = 80 °C; [styrene] = 0.2 mmol; [H_2_O_2_] = 1.0 mmol; acetonitrile = 3 mL.

To obtain kinetic order with respect to the pre-catalyst, the amount of substrate, the concentration of oxidant (H_2_O_2_), the volume of solvent and reaction temperature were kept constant while varying the concentration of the pre-catalyst. The results presented in Figure 7 show that the initial reaction rate increased linearly with catalyst concentration, amounting to a first-order dependence which agrees with established conditions for the selective oxidation of styrene to benzaldehyde by a Cr-ZSM-5 catalyst [43,47].

**Figure 8:**
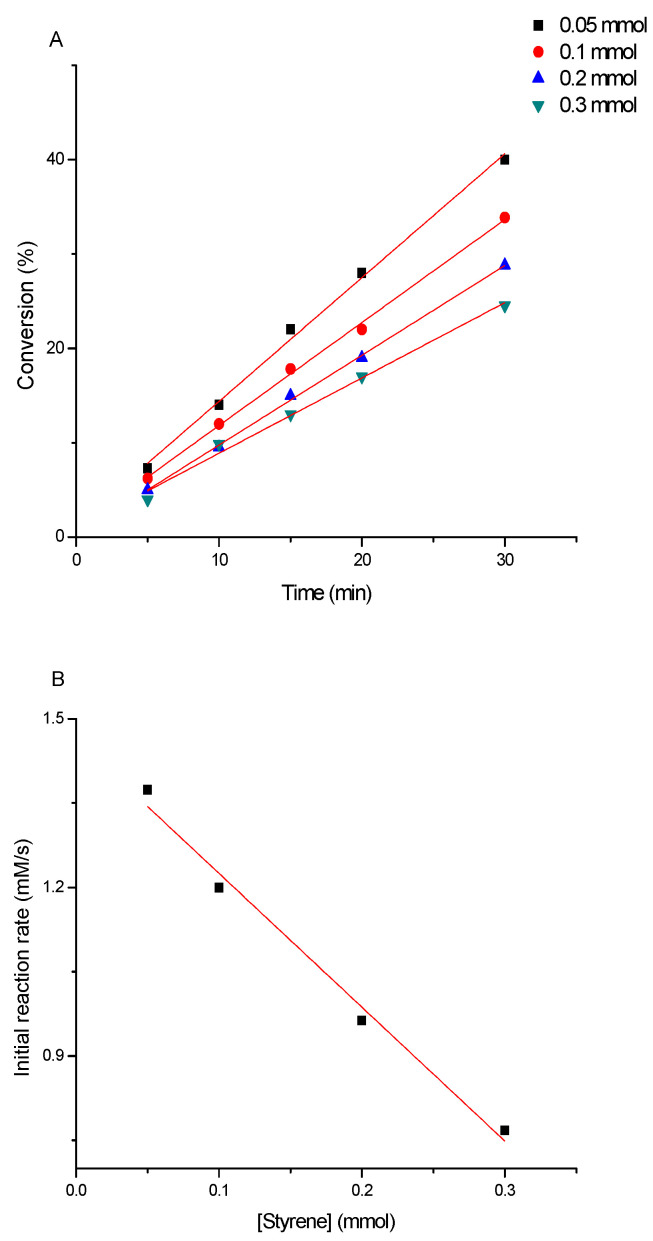
(**A**) Influence of varying styrene concentration with time; (**B**) plot of initial reaction rate vs styrene concentration. Reaction conditions: T = 80 °C; [catalyst] = 0.002 mmol; [H_2_O_2_] = 1 mmol; [acetonitrile] = 3 mL.

However, when the substrate concentration was varied while other parameters were kept constant, as expected, the kinetic order of the reaction was observed to decrease with an increase in styrene concentration. With catalyst concentration kept constant, an increase in styrene concentration led to a dilution effect that negatively affected the availability of the metal centre, thereby leading to the observed decrease in the conversion of styrene (Figure 8) [44,48].

**Figure 9:**
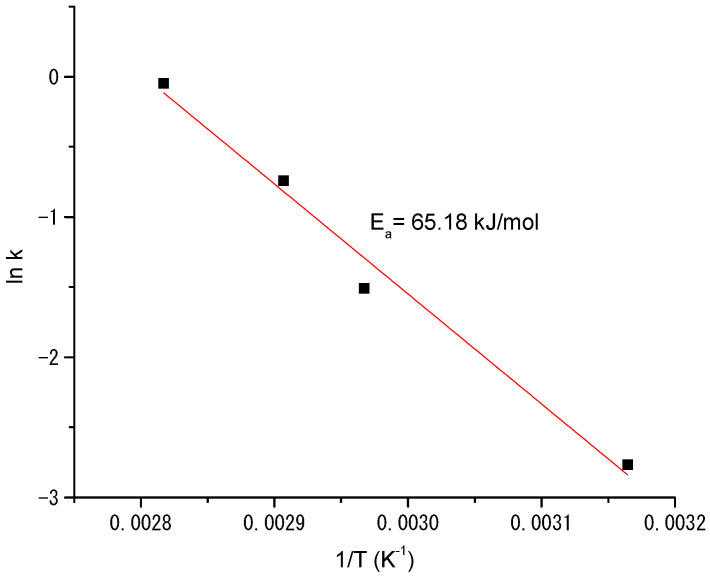
Arrhenius plot of the pseudo-first-order rate kinetic constants for styrene oxidation.

The activation energy was determined by varying the reaction temperature from 43 °C to 82 °C, the solvent’s (acetonitrile) boiling temperature. A first-order kinetic constant was calculated, and the results were fitted to the Arrhenius equation and plotted in Figure 9. The activation energy of 65 ± 3 kJ/mol was calculated for the homogeneous process, much higher than the 15 kJ/mol reported for a heterogeneous Cr-ZSM-5 catalyst [47]. The efficiency of the Ni(II) catalyst is illustrated when the bond dissociation energy of 614 kJ/mol for a C=C bond is considered. High activation energy usually implies that potentially harsh conditions are required to activate the reaction [49]. The efficiency of the Ni(II) catalysts reported herein meant that the reaction was still achievable under relatively mild conditions.

### 3.4. UV Study of the Reaction

**Figure 10:**
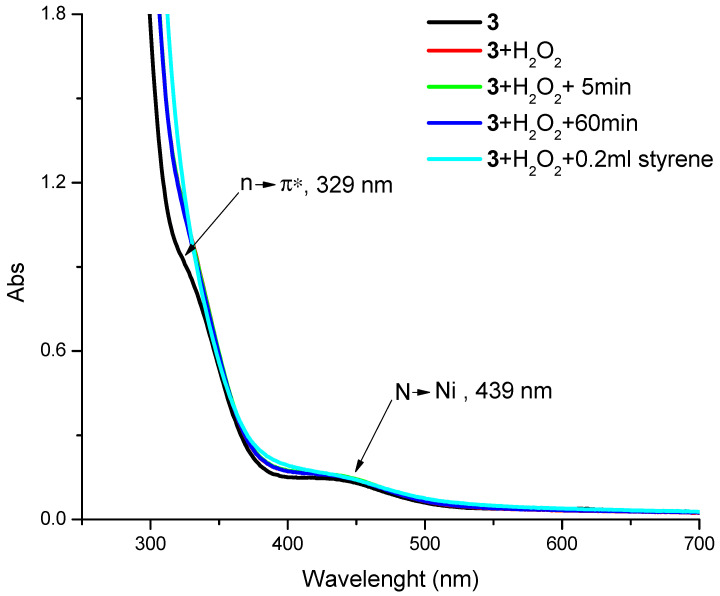
UV-visible analysis of the oxidation of styrene catalyzed by complex **3**, using H_2_O_2_ as the oxidant.

Upon review of related literature, it is clear that the generation of a Ni(III) intermediate is possible in the catalytic oxidation of styrene [14]. Thus, to understand the mechanism of the current systems, the absorption spectrum of complex **3** was monitored during the reaction (Figure 10). The UV spectra showed a shoulder at 329 nm assigned to the n-π* intra-ligand charge transfer band and a signal at 439 nm assigned to N→Ni charge transfer [50,51]. On adding the oxidant, the shoulder band at 329 flattens while the signal at 439 nm remained unperturbed even after 1 h. After adding the substrate, the spectrum pattern remained unchanged even with the reaction time extended beyond 6 h. Based on these observations, it is proposed that there is no clear indication of Ni(II) oxidation to Ni(III) on the addition of the oxidant. The Ni(II) centre activates the oxidation of the substrate. The UV data indicate a relatively stable metal complex under the oxidation reaction conditions, with the catalytic process mostly driven through a metal–ligand cooperative process. Furthermore, the persistence of the absorbance at 439 nm even after the addition of the oxidant ruled out possible hydrolysis of the imine functionality or hemilability of the N-Ni bond during the catalytic cycle [52]. However, the inactivation of the catalyst beyond 6 h might be due to possible catalyst degradation as observed in similar imine-containing complex catalysts [53,54].

### 3.5. Cyclic Voltammetry (CV) Study

**Figure 11:**
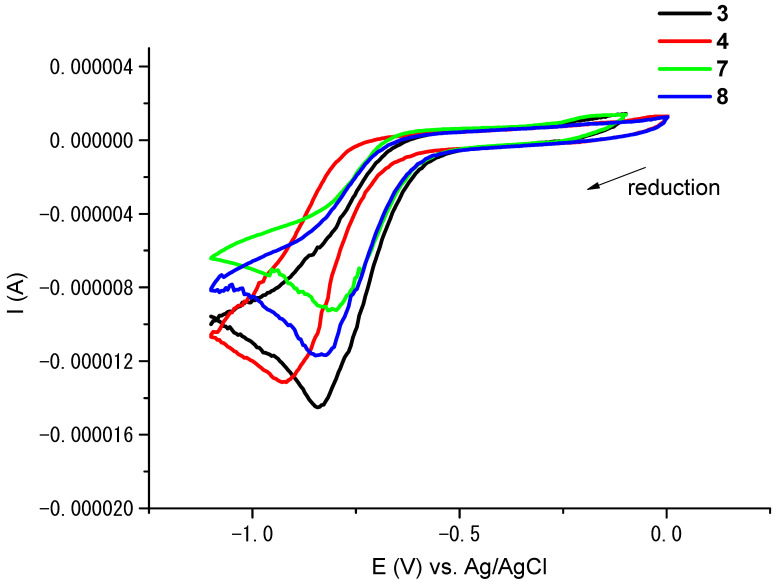
Cyclic voltammograms of nickel complexes, **3** (black), **4** (red), **7** (green) and **8** (blue) in DMSO.

A CV experiment was performed to obtain further insights into the oxidation/reduction properties of the Ni(II) complexes involved in the catalytic process (Figure 11). Irreversible one-electron reduction peaks were observed for all the complexes at −0.84, −0.93, −0.79 and −0.83 V for complexes **3**, **4**, **7** and **8**, respectively. This was attributed to the reduction of Ni(II) to Ni(I) for all the complexes [55]. However, by comparing with similar Ni(II) square planar complexes of a tetradentate amide-based ligand with reduction potentials of −1.8 to −2.4 V [22], complexes **3**, **4**, **7** and **8** are on the higher side, signifying that they are easily reduced. However, no oxidation peak was observed on reverse scanning, implying that there is no possibility of a reversible Ni(I)/(II) as well as Ni(II) to Ni(III) redox cycle in these catalyst systems. This is well in agreement with the UV data (Figure 10), indicating a relatively stable ligand–metal environment for complex **3** under oxidizing conditions.

## 4. Conclusions

We have described the synthesis of four new Schiff base functionalized 1,2,3-triazolylidene nickel complexes. Square planar coordination geometry was proposed for all the nickel complexes, and all complexes were efficient in the oxidation of styrene to benzaldehyde with good conversions and high selectivities. The kinetics of the reaction was first order in catalyst, fractional order in oxidant, and the activation energy was determined to be 65 ± 3 kJ/mol.

## Figures and Tables

**Table 1:**
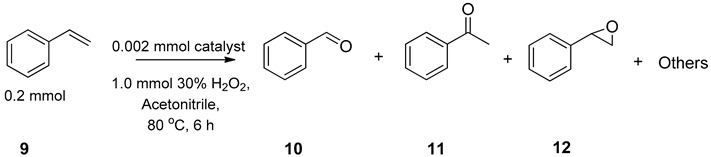
Oxidation of styrene to its oxygenates catalyzed by various nickel catalysts ^a^.

Entry	Catalyst	Time (h)	Conversion (%) ^b^	^c^ Selectivity (%)				TON ^d^	TOF ^e^
				Benzaldehyde	Acetophenone	Styrene Oxide	Others		
**1**	H_2_O_2_	6	22	97	-	-	3	-	-
**2**	**3** (no H_2_O_2_ added)	6	-	-	-	-	-	-	-
**3**	**3**	3	77	74	18	3	5	770	260
**4**	**3**	6	88	70	22	3	5	880	150
**5**	Ni(diglyme)Cl_2_	6	35	93	-	-	7	350	58
**6**	**1**	6	26	94	-	-	6	260	43
**7**	**4**	3	62	91	1	5	3	620	210
**8**	**4**	6	75	90	1	6	3	750	125
**9**	**7**	3	49	92	1	5	2	490	160
**10**	**7**	6	65	90	0	6	4	650	110
**11**	**8**	3	50	94	1	5	0	500	170
**12**	**8**	6	69	91	1	5	3	690	110

^a^ Except otherwise mentioned, all reactions where conducted with: styrene (substrate, 0.2 mmol), catalyst (0.002 mmol), 30% H_2_O_2_ (oxidant, 1.0 mmol), acetonitrile (solvent, 3 mL), 80 °C, 6 h. Yield is based on GC conversion, an average of two runs. ^b^ Conversion = conc. products/initial conc. styrene × 100. ^c^ Selectivity = percentage of benzaldehyde, acetophenone, styrene oxide and other side products. ^d^ TON = the ratio of moles of the product obtained to the moles of the catalyst. ^e^ TOF = TON/h (as h^−1^).

**Table 2:** Comparison of homogeneous catalytic activities of nickel complexes in the oxidation of styrene.

Entry	Catalyst	Oxidant	Time (h)	Conversion (%)	Selectivity (%)			Ref.
					Benzaldehyde	Styrene Oxide	Others	
**1**	**3**	H_2_O_2_	3	77	74	2	24	This work
**2**	**3**	H_2_O_2_	6	88	70	3	27	This work
**3**	NiLa ^a^	TBHP	6	58	49	9	-	[17]
**4**	NiLb ^b^	H_2_O_2_	4	57	26	14	-	[18]
**5**	NiH_2_ID ^c^	H_2_O_2_	3	52	-	61	-	[14]

^a^ NiLa, La = 2,2*′*-((1E,1E)-((2,2-dimethylpropane-1,3-diyl)bis(azanylylidene))bis(propan-1-yl-1-ylidene))diphenol. ^b^ NiLb, Lb = N,N*′*-bis(3-methoxy-salicylid-ene-ethylene-1,2-diamine. ^c^ NiH_2_ID, H_2_ID = dihydroindolone.

## Data Availability

Not applicable.

## Supplementary Materials

The following supporting information can be downloaded at: https://www.mdpi.com/article/10.3390/molecules27154941/s1, Figures S1–S20: NMR data for all the compounds and selected GC data for the catalysis.
