# Supplementary material for: Oxidation of Styrene to Benzaldehyde Catalyzed by Schiff Base Functionalized Triazolylidene Ni(II) Complexes
Source: Molecules. 2022 Aug 3;27(15):4941. doi: 10.3390/molecules27154941 (PMC9370366; doi:10.3390/molecules27154941)
Supplement: Supplementary file 1 [file molecules-27-04941-s001.zip › molecules-1761276-supplementary.pdf]

Oxidation of styrene to benzaldehyde catalysed by Schiff base-functionalised triazolylidene Ni(II) complexes

Nasir S. Lawal, Halliru Ibrahim, and Muhammad D. Bala*

General supporting information

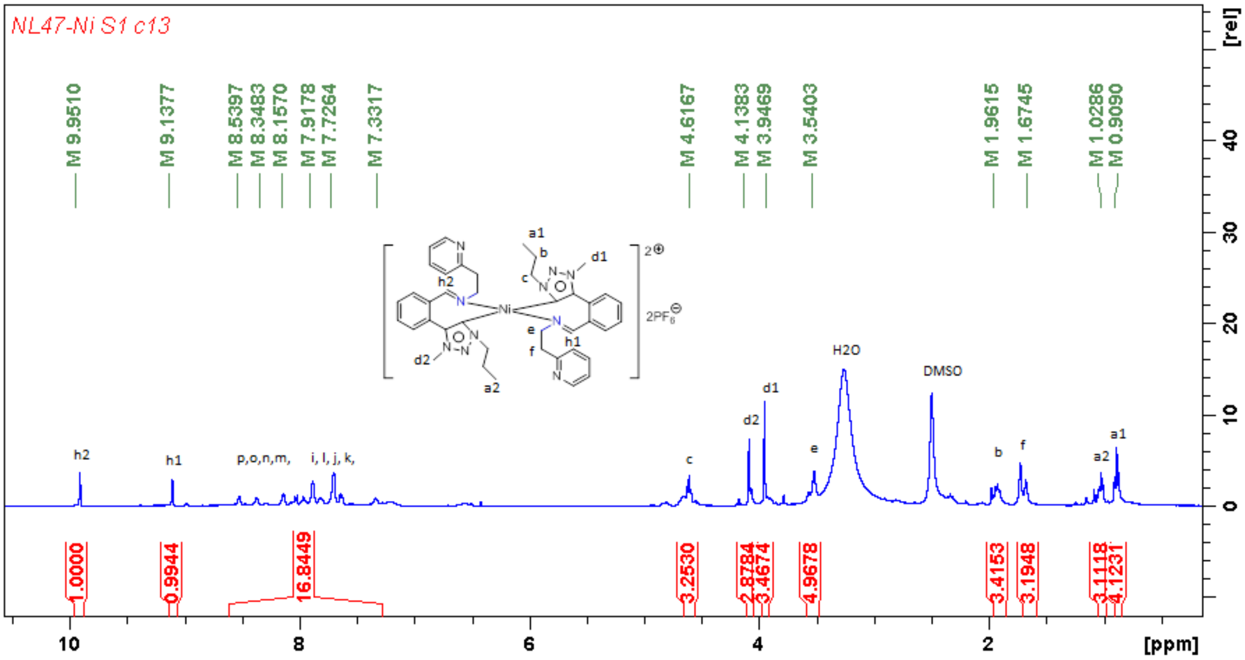

Figure S1: ^{1}H NMR of Ni complex **3**

*Correspondence to: e-mail- bala@ukzn.ac.za

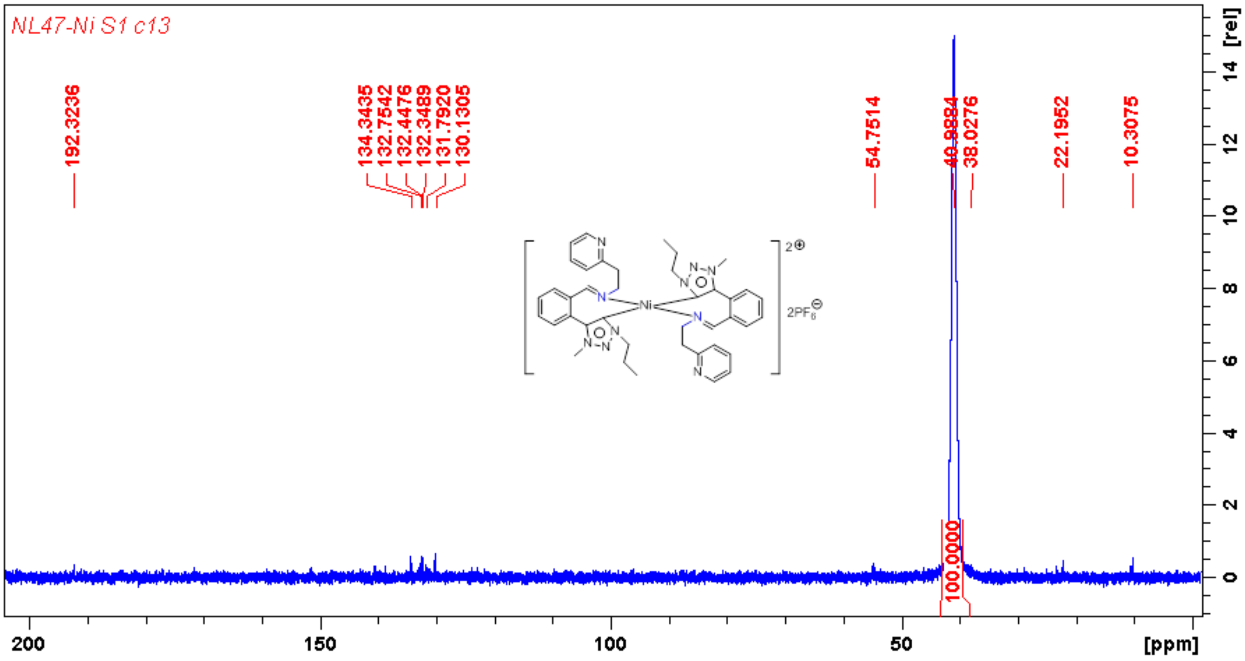

Figure SI 2: ^{13}C NMR of Ni complex **3**

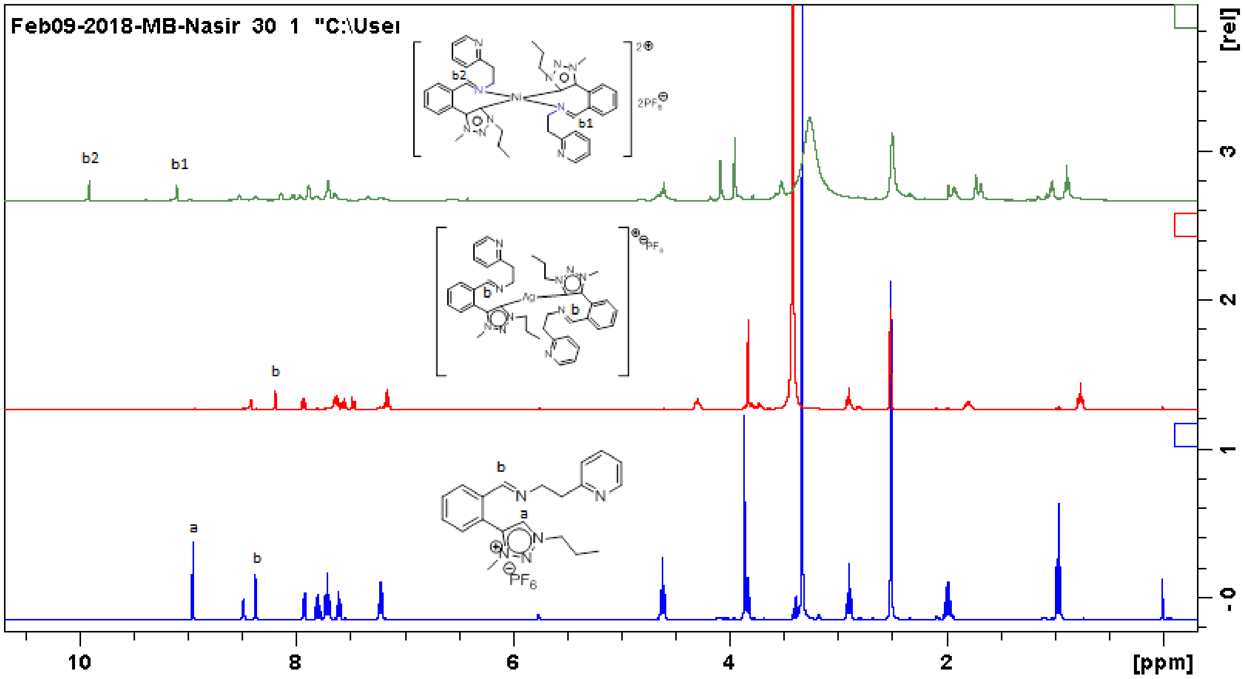

Figure SI 3: ^{1}H NMR showing the disappearance of carbene proton from ligand precursor (blue line, position a at 8.9 ppm) to silver complex (red line) and on formation of the nickel complex **3** (green line) appearance of the splitting of the two imine protons (position b1 and b2) due to different coordination environment.

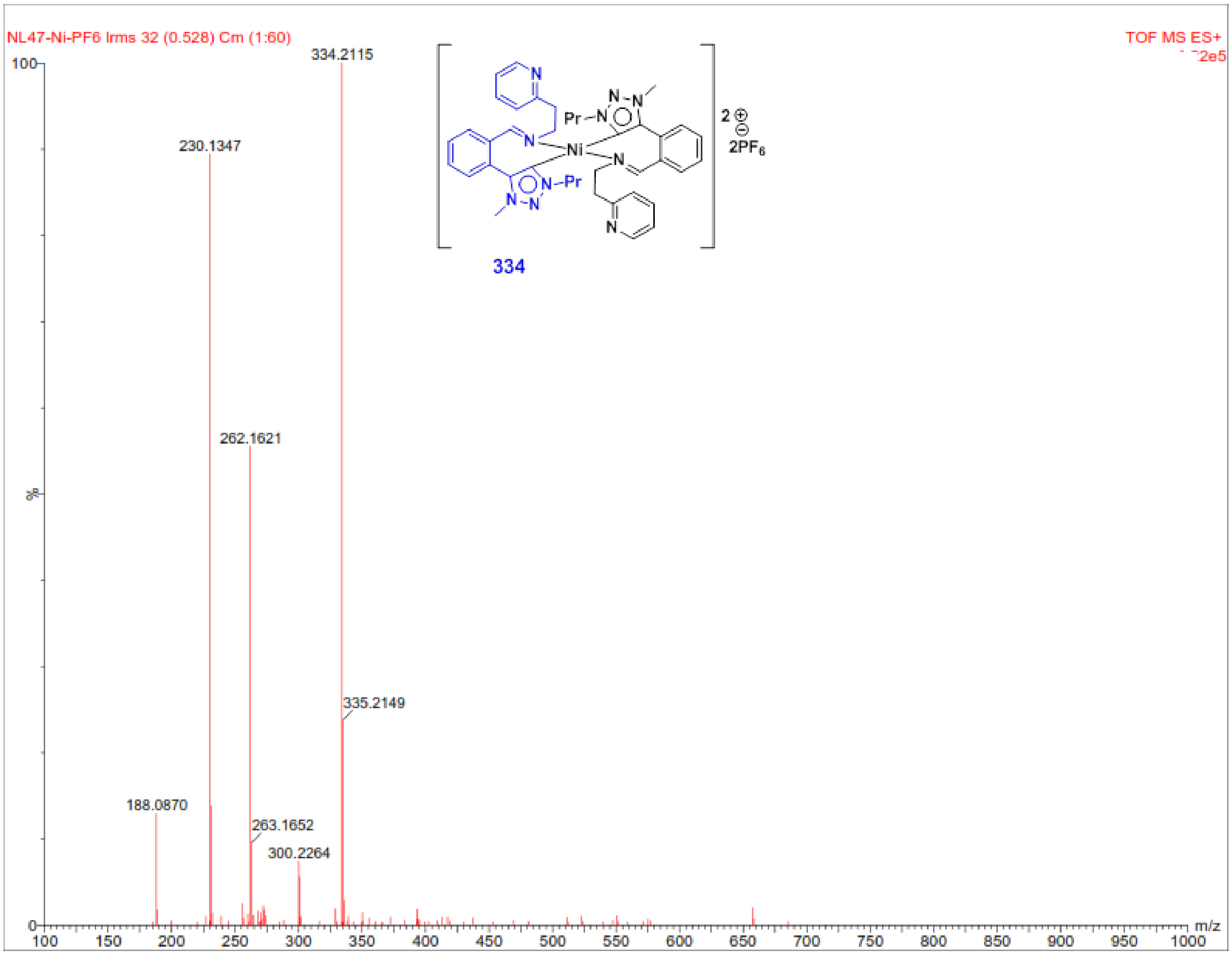

Figure SI 4: LRMS of Ni complex **3**

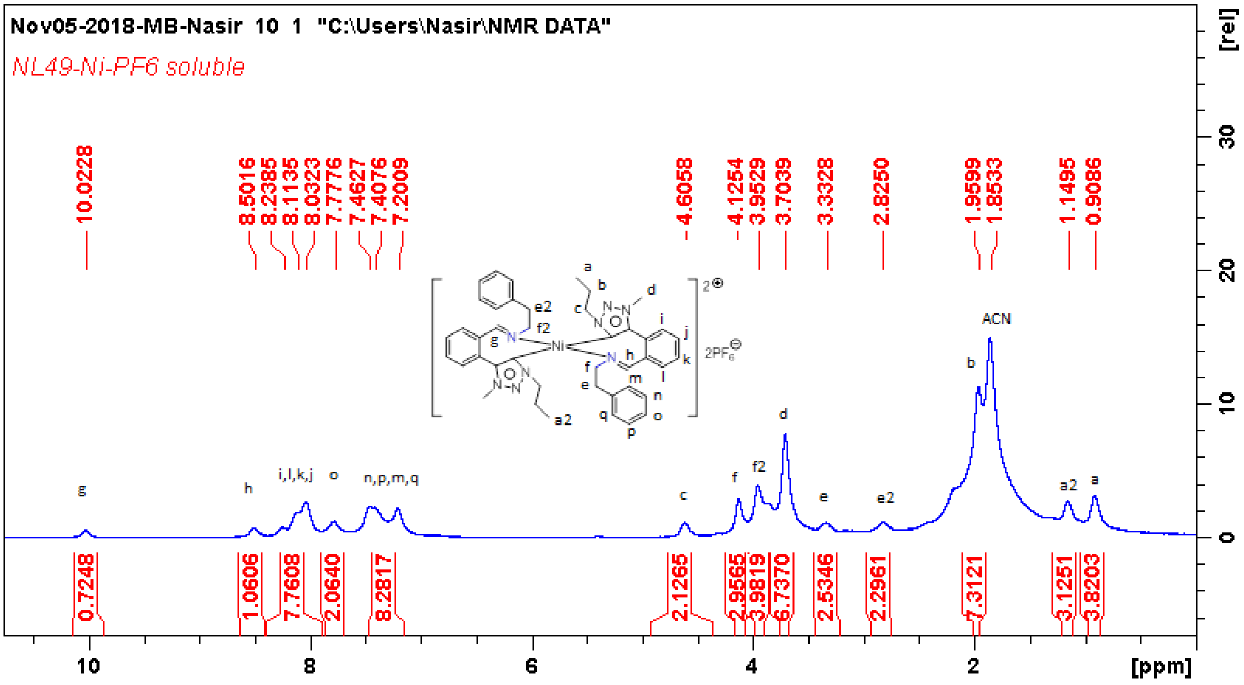

Figure SI 5: ^{1}H NMR of Ni complex **4**

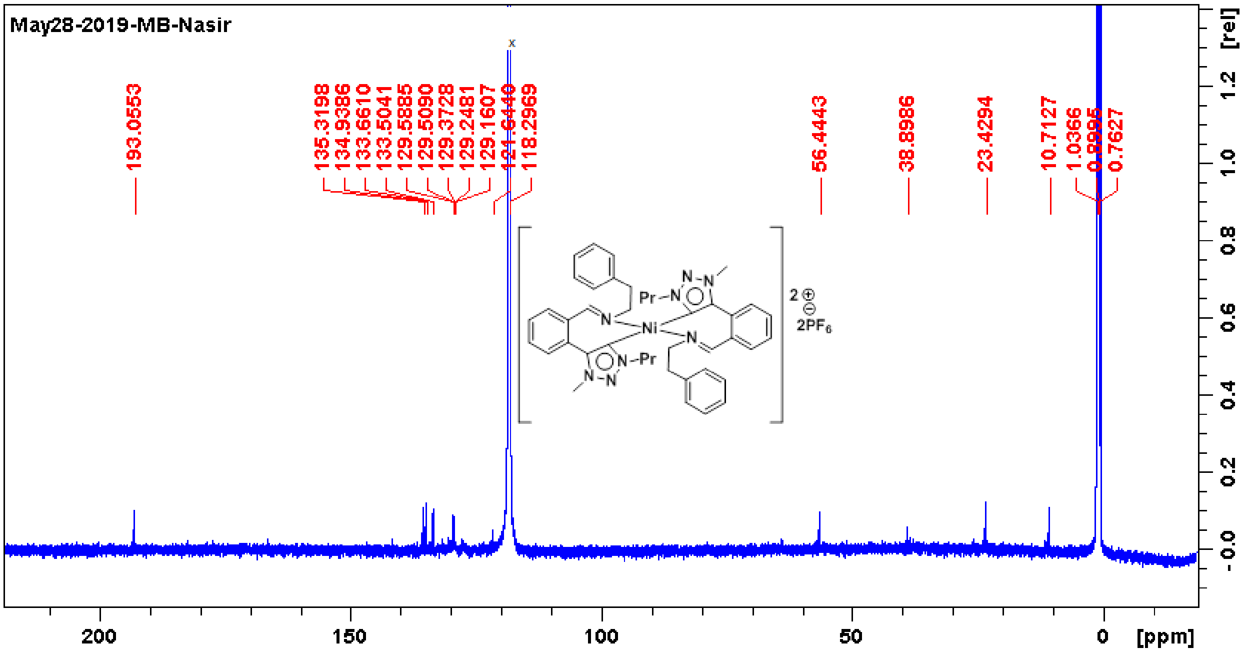

Figure SI 6: ^{13}C NMR of Ni complex **4**

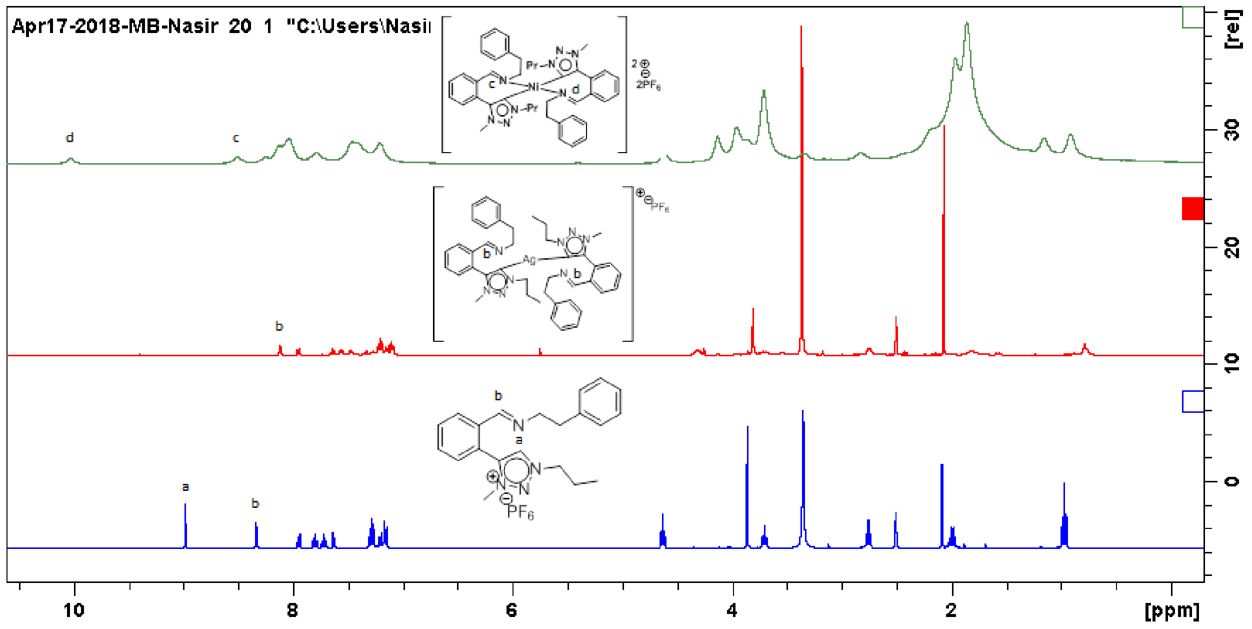

Figure SI 7: ^{1}H NMR showing the disappearance of carbene proton from ligand precursor (blue line, position a at 8.9 ppm) to silver complex (red line) and on formation of the nickel complex **4** (green line) appearance of the splitting of the two imine protons (position h1 and h2) due to different coordination environment.

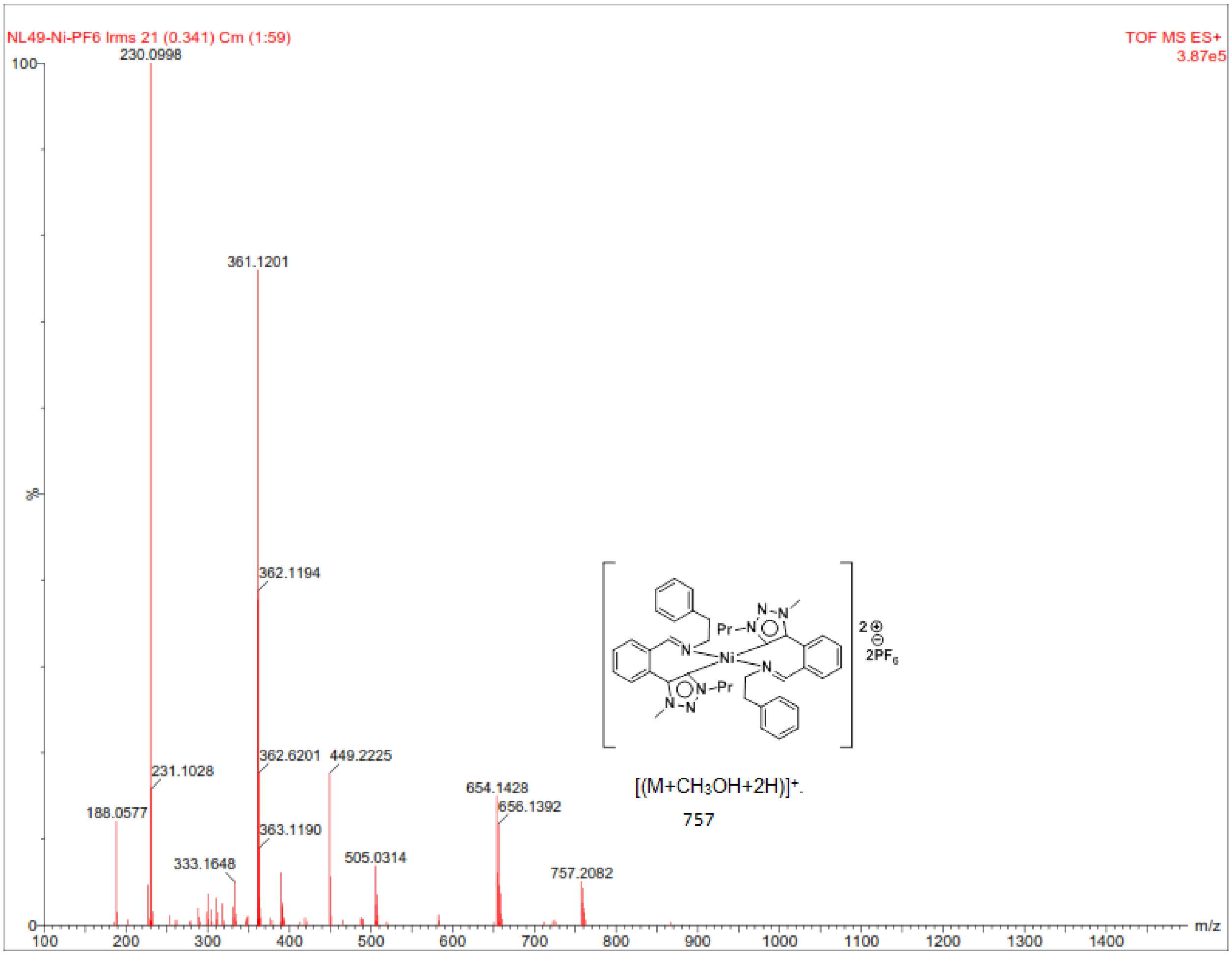

Figure SI 8: LRMS of Ni complex **4**

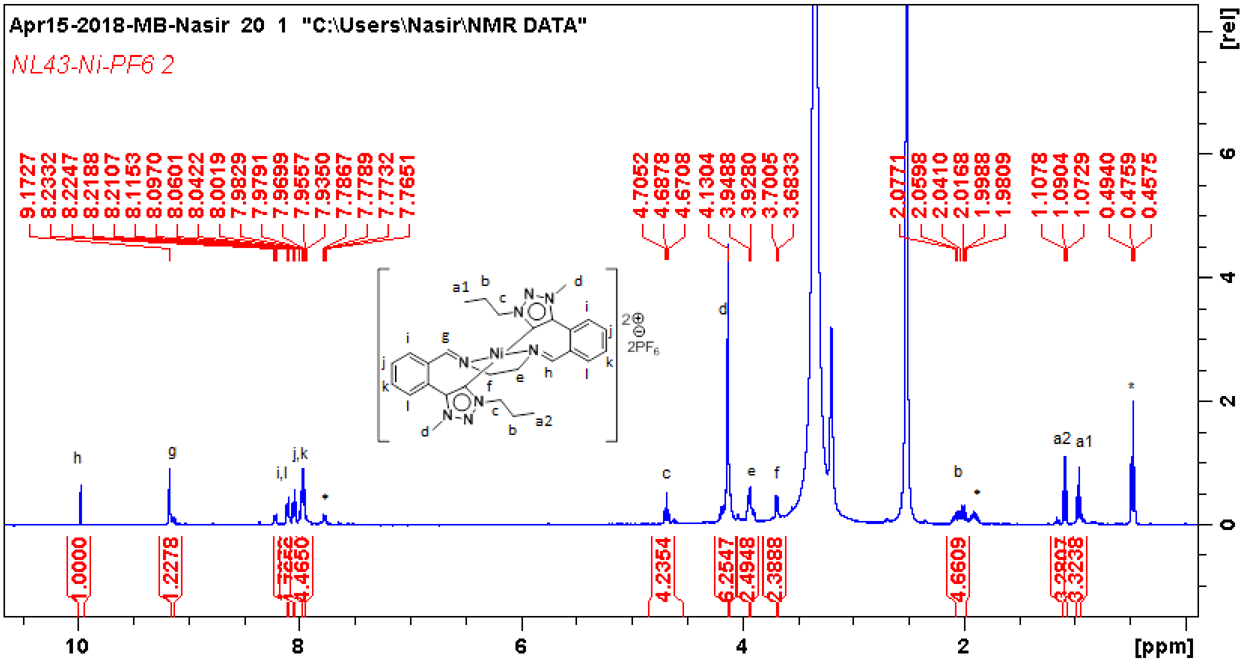

Figure SI 9: ^{1}H NMR of Ni complex **7**

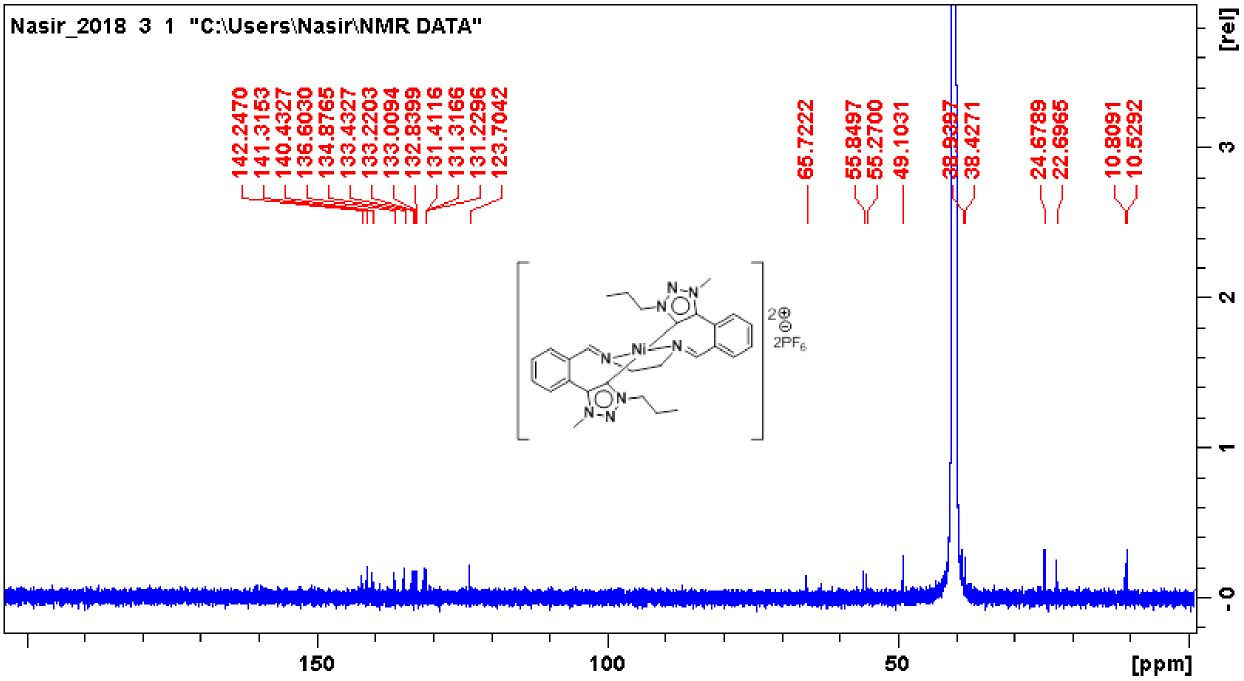

Figure SI 10: ^{13}C NMR of Ni complex **7**

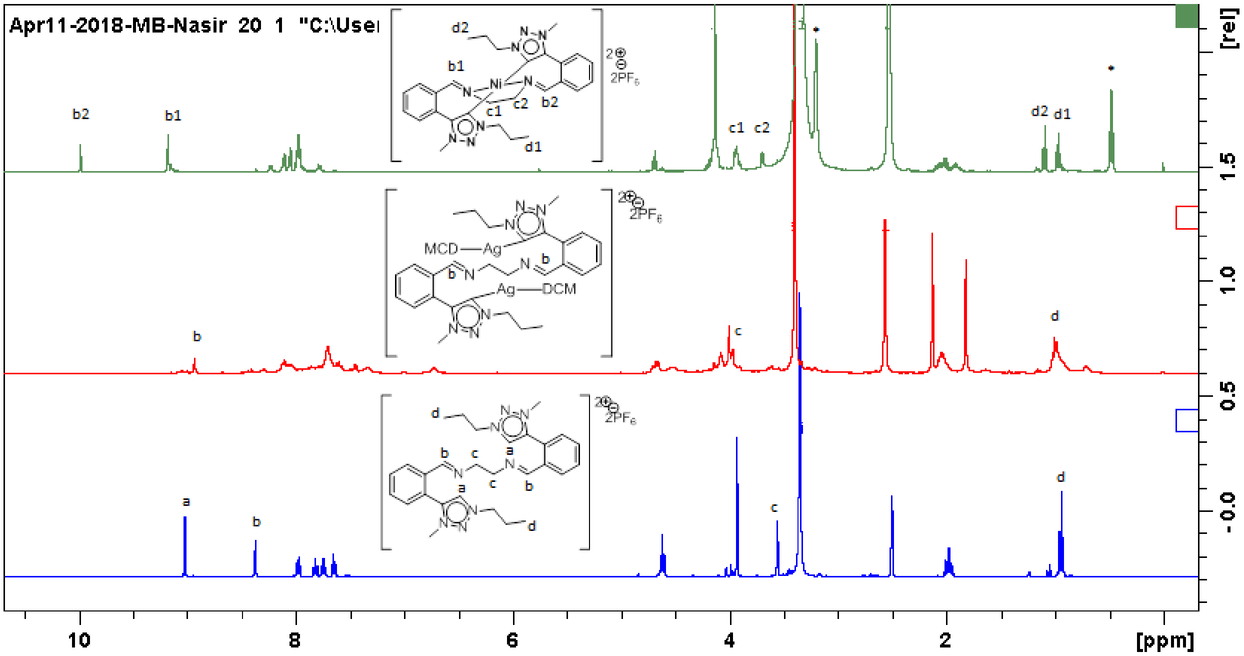

Figure SI 11: ^{1}H NMR showing the disappearance of carbene proton from ligand precursor (blue line, position a at 8.9 ppm) to silver complex (red line) and on formation of the nickel complex **7** (green line) appearance of the splitting of the two imine protons (position b1 and b2) due to different coordination environment

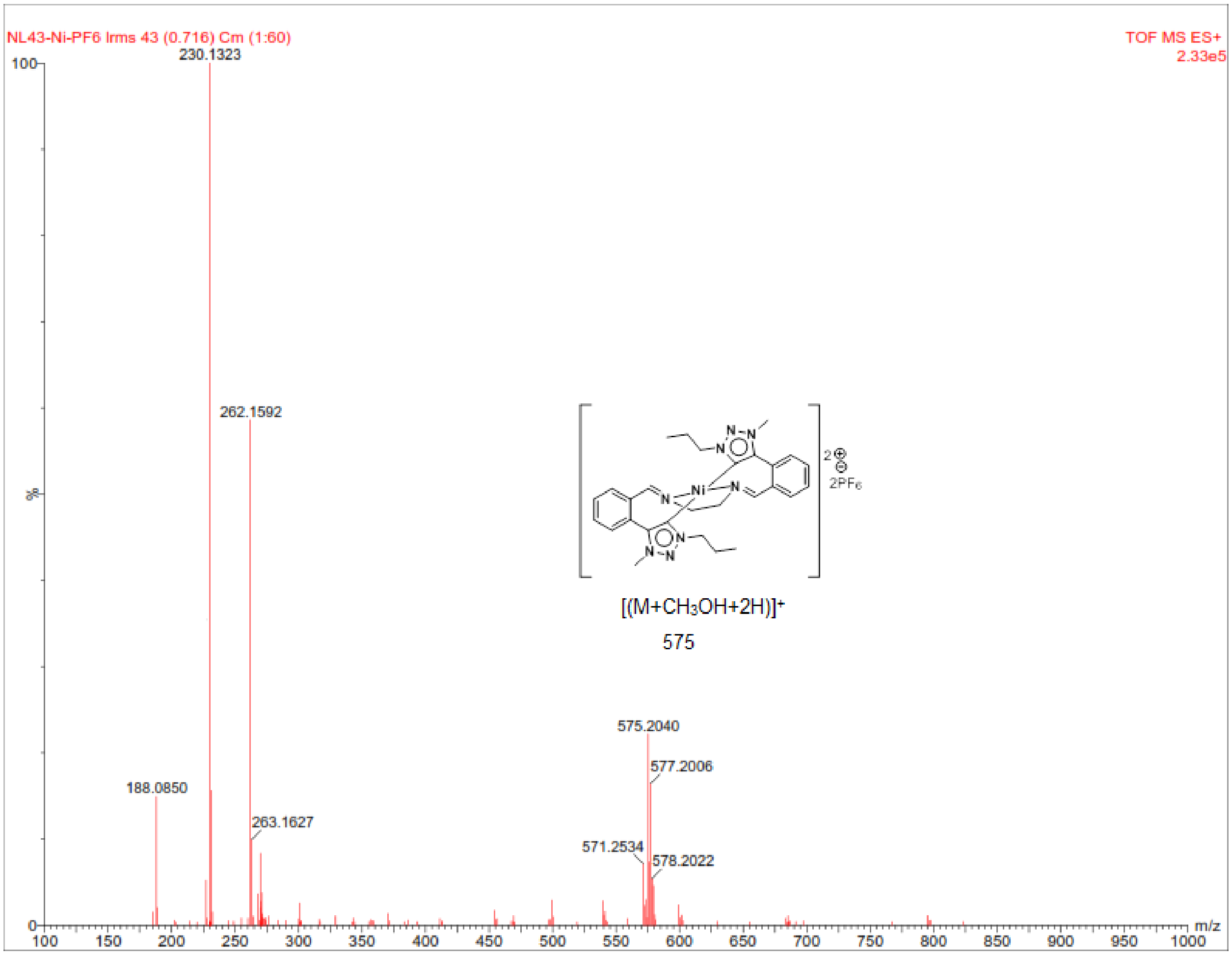

Figure SI 12: LRMS of Ni complex **7**

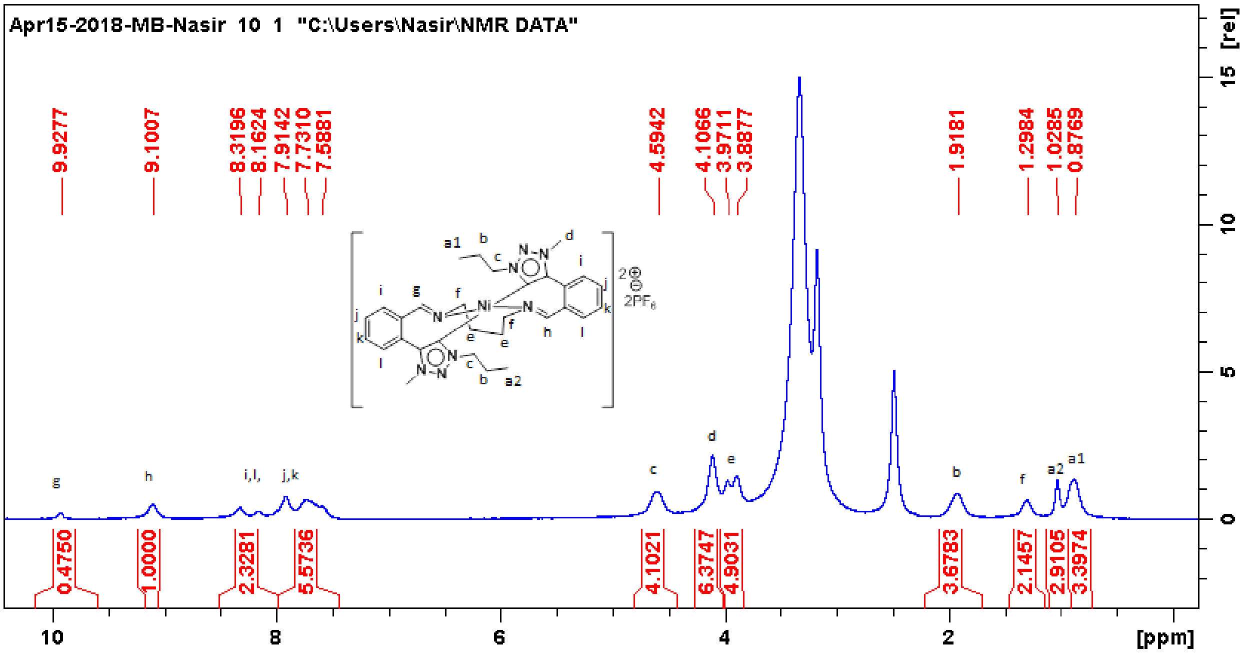

Figure SI 13: ^{1}H NMR of Ni complex **8**

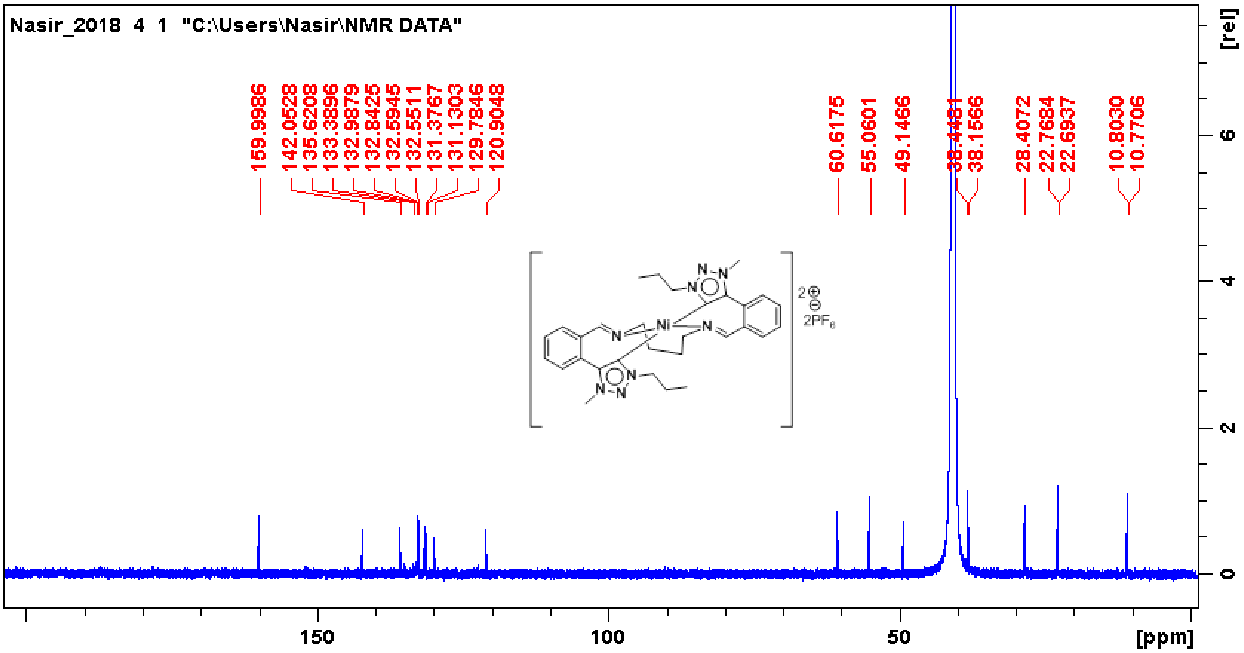

Figure SI 14: ^{13}C NMR of Ni complex **8**

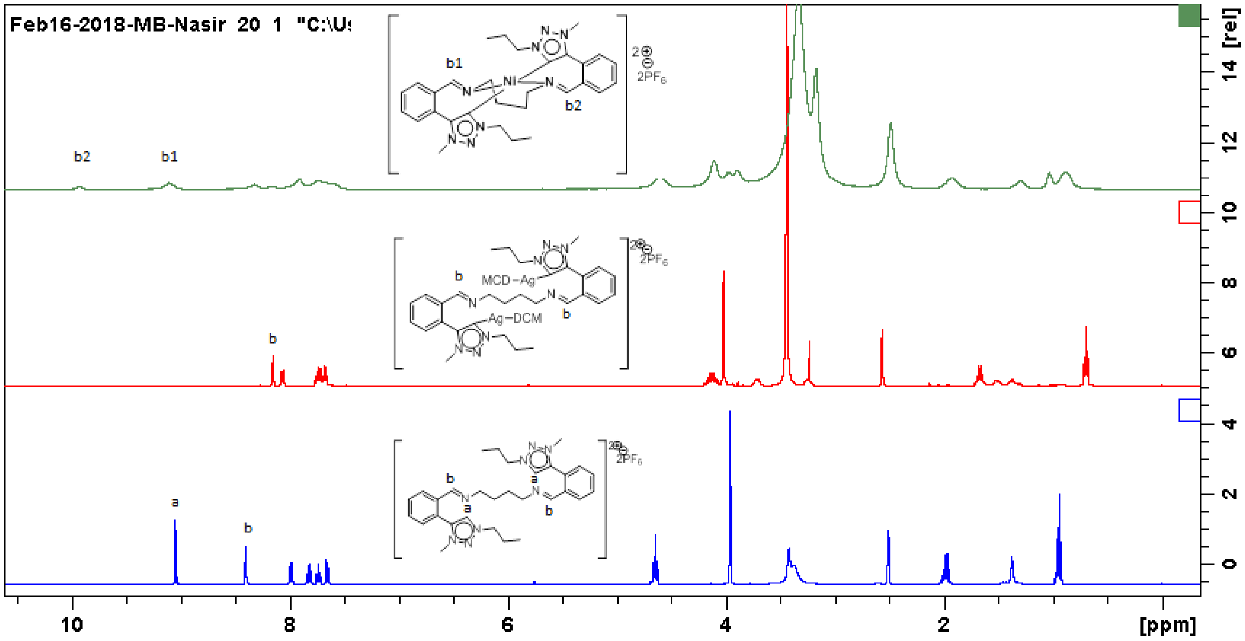

Figure SI 15: ^{1}H NMR showing the disappearance of carbene proton from ligand precursor (blue line, position a at 8.9 ppm) to silver complex (red line) and on formation of the nickel complex **8** (green line) appearance of the splitting of the two imine protons (position b1 and b2) due to different coordination environment.

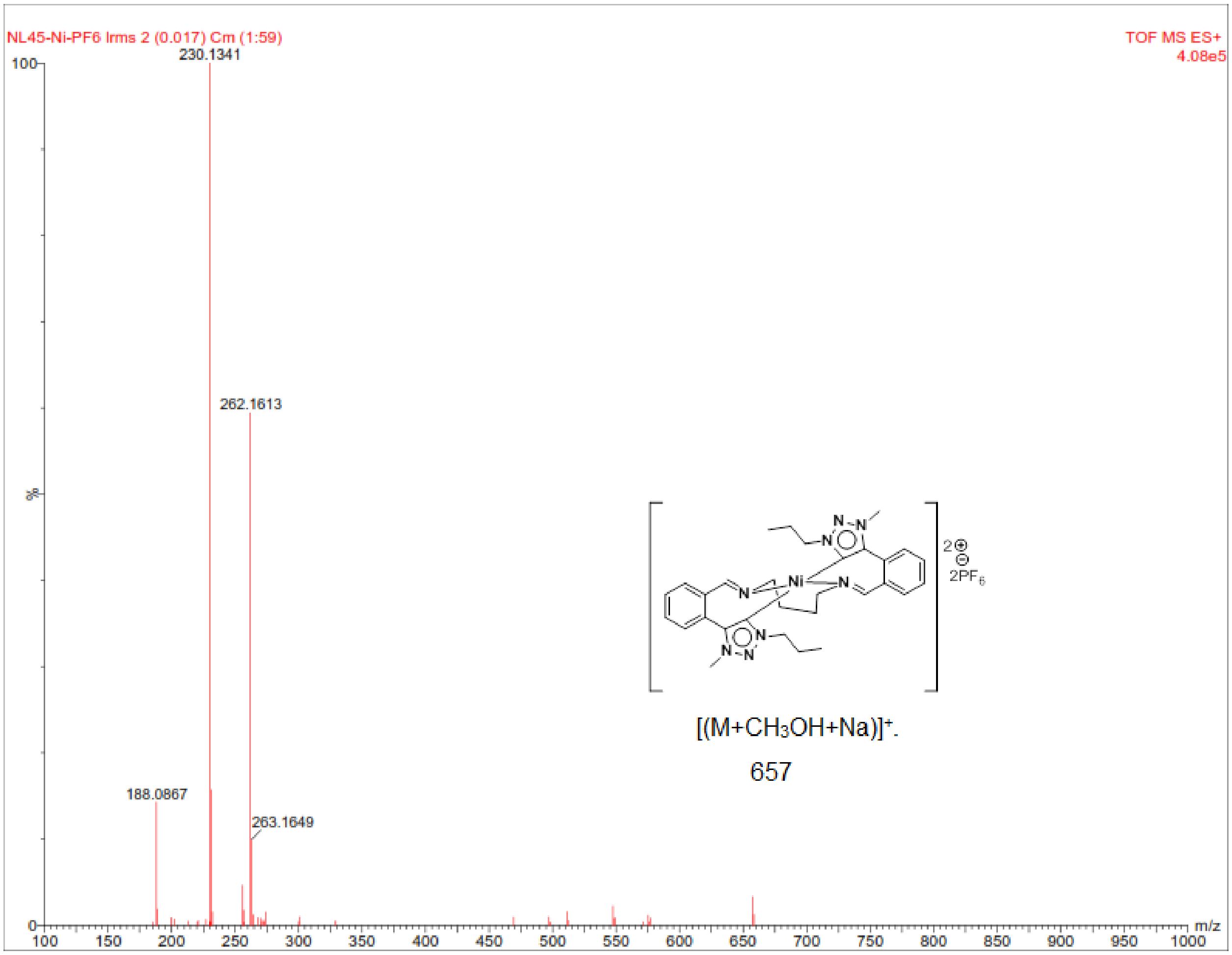

Figure SI 16: LRMS of Ni complex **8**

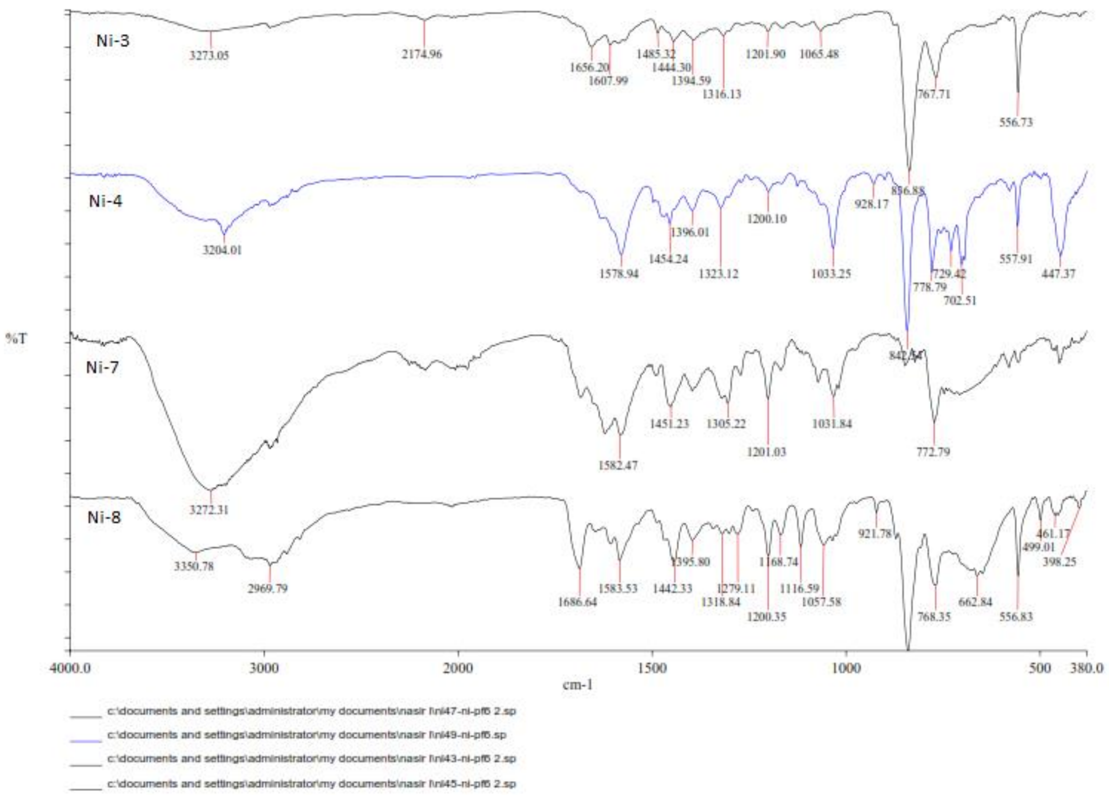

Figure SI 17: Stacked IR spectra of Ni complexes **3**-**4** and **7**-**8**

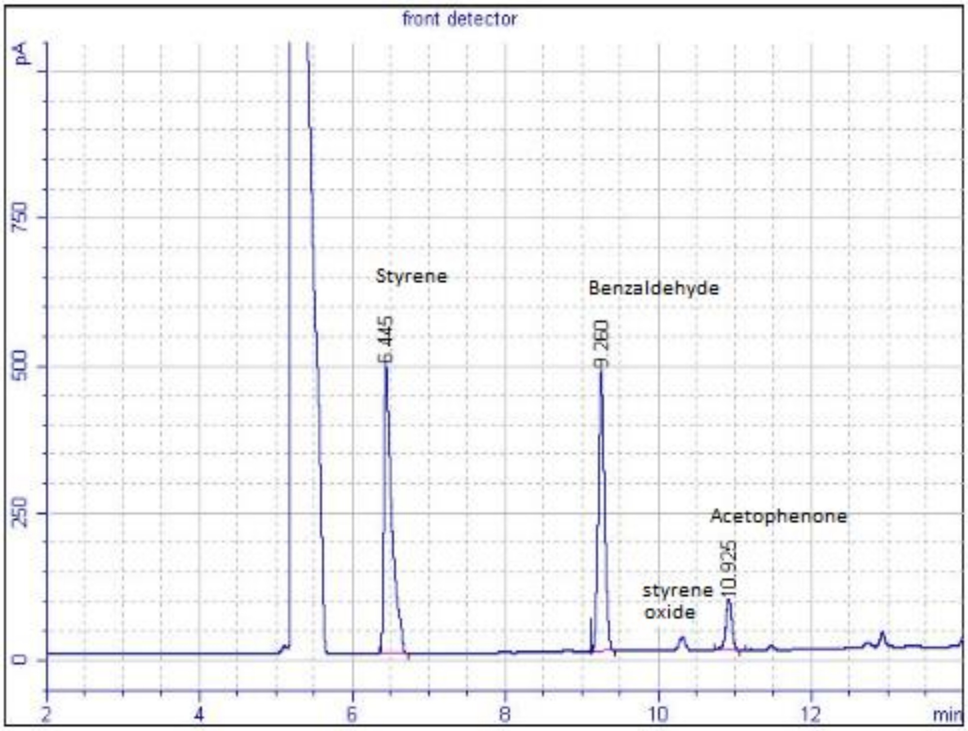

Figure SI 18: Chromatogram for the oxidation of styrene catalyzed by Ni complex **3** after 1 hour.

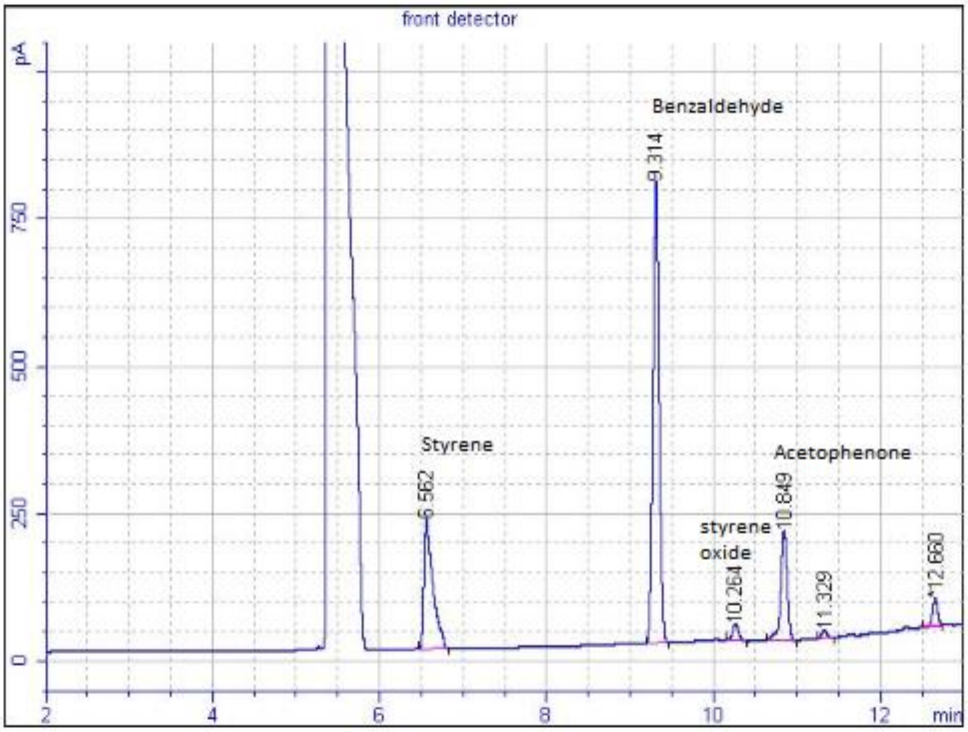

Figure SI 19: Chromatogram for the oxidation of styrene catalyzed by Ni complex **3** after 3 hours.

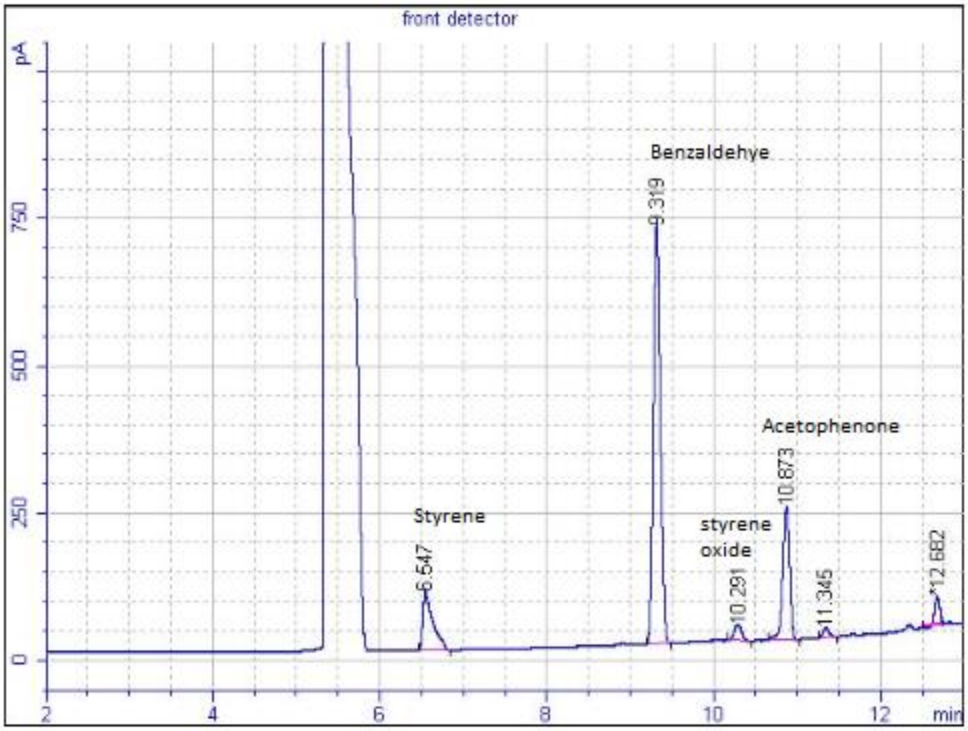

Figure SI 20: Chromatogram for the oxidation of styrene catalyzed by Ni complex **3** after 6 hours
